# Transposon‐driven transcription is a conserved feature of vertebrate spermatogenesis and transcript evolution

**DOI:** 10.15252/embr.201744059

**Published:** 2017-05-12

**Authors:** Matthew P Davis, Claudia Carrieri, Harpreet K Saini, Stijn van Dongen, Tommaso Leonardi, Giovanni Bussotti, Jack M Monahan, Tania Auchynnikava, Angelo Bitetti, Juri Rappsilber, Robin C Allshire, Alena Shkumatava, Dónal O'Carroll, Anton J Enright

**Affiliations:** ^1^ European Molecular Biology LaboratoryEuropean Bioinformatics Institute (EMBL‐EBI)CambridgeUK; ^2^ European Molecular Biology LaboratoryMouse Biology OutstationMonterotondoItaly; ^3^ MRC Centre for Regenerative MedicineInstitute for Stem Cell Research, School of Biological Sciences, University of EdinburghEdinburghUK; ^4^ Institut Pasteur – Bioinformatics and Biostatistics HubC3BI, USR 3756 IP CNRSParisFrance; ^5^ Wellcome Trust Centre for Cell BiologySchool of Biological SciencesUniversity of EdinburghEdinburghUK; ^6^ Institut Curie – CNRS UMR3215INSERM U934ParisFrance; ^7^ Institute of BiotechnologyTechnische Universität BerlinBerlinGermany

**Keywords:** endogenous retroviruses, genome evolution, lncRNA, spermatogenesis, transcriptome, Chromatin, Epigenetics, Genomics & Functional Genomics, Transcription

## Abstract

Spermatogenesis is associated with major and unique changes to chromosomes and chromatin. Here, we sought to understand the impact of these changes on spermatogenic transcriptomes. We show that long terminal repeats (LTRs) of specific mouse endogenous retroviruses (ERVs) drive the expression of many long non‐coding transcripts (lncRNA). This process occurs post‐mitotically predominantly in spermatocytes and round spermatids. We demonstrate that this transposon‐driven lncRNA expression is a conserved feature of vertebrate spermatogenesis. We propose that transposon promoters are a mechanism by which the genome can explore novel transcriptional substrates, increasing evolutionary plasticity and allowing for the genesis of novel coding and non‐coding genes. Accordingly, we show that a small fraction of these novel ERV‐driven transcripts encode short open reading frames that produce detectable peptides. Finally, we find that distinct ERV elements from the same subfamilies act as differentially activated promoters in a tissue‐specific context. In summary, we demonstrate that LTRs can act as tissue‐specific promoters and contribute to post‐mitotic spermatogenic transcriptome diversity.

## Introduction

**Figure 1:**
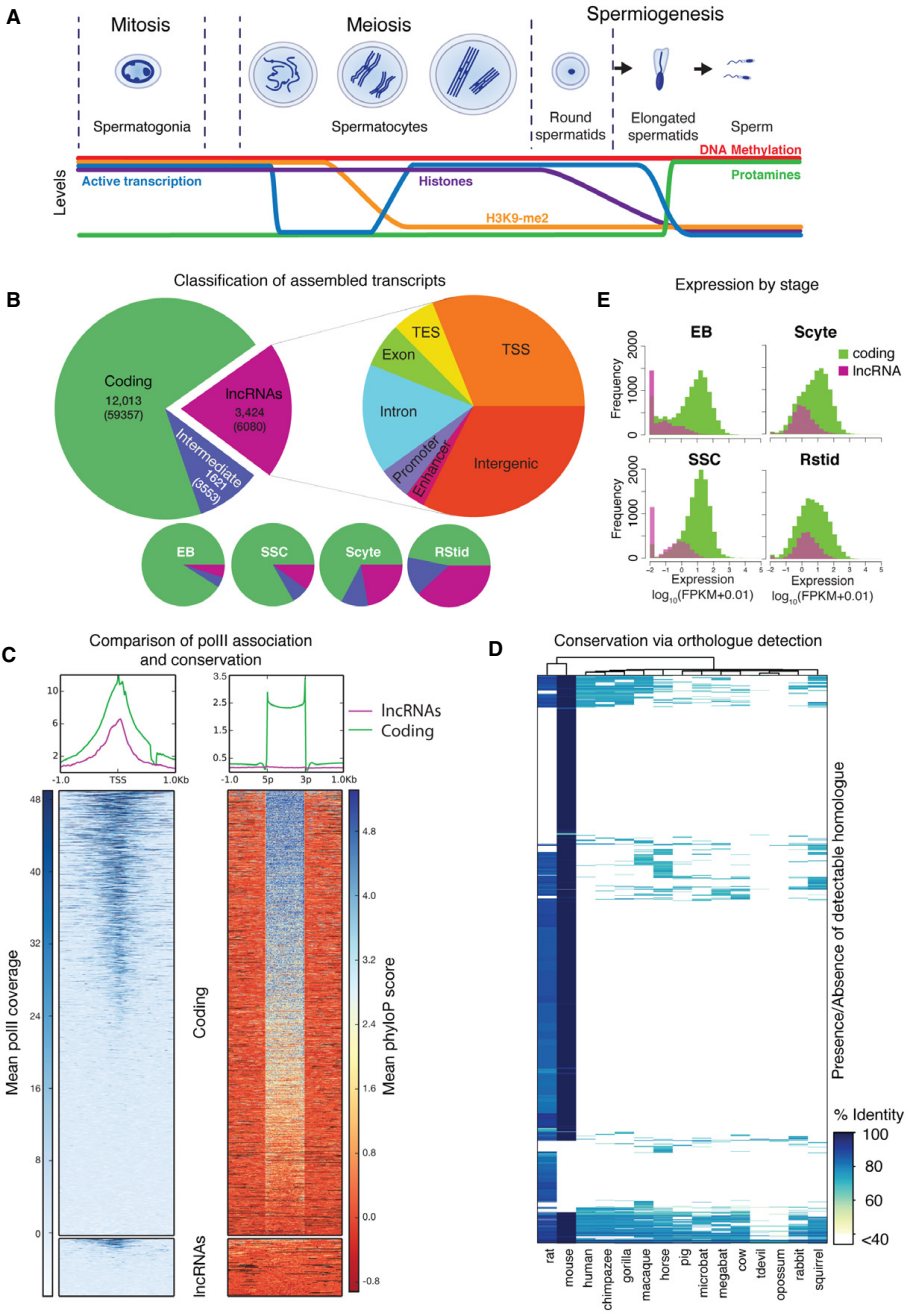
Discovery and analysis of non‐coding transcripts during murine spermatogenesis The stages and major events of murine spermatogenesis.Left: The number of detected transcript clusters for protein‐coding (green), lncRNA (purple) and intermediate classes (i.e. transcripts which passed one of two tests for coding potential). In each case, the total number of detected non‐redundant transcripts is shown in parentheses. Right: The genomic context classification for the detected lncRNA clusters, broken down according to hierarchy (see Appendix Supplementary Methods). Bottom: The fraction of detected transcript clusters of each type according to the cell type in which they are most highly expressed.Left bottom: Testes‐specific Pol II occupancy at TSSs (± 1 kb) from the assembly for coding and lncRNA transcripts. Left top: Averaged Pol II occupancy profiles around TSSs for coding (green) and lncRNAs (purple). Right bottom: Scaled exon conservation levels (± 1 kb) for coding and lncRNA transcripts. Right top: Averaged exon conservation summary profiles for coding (green) and lncRNAs (purple).Heatmap showing percentage sequence identity for each of 2,002 detected mouse lncRNA transcripts against identified matches in the complete genomes of 15 species. The lowest level of detectable homology was 40% (white). A total of 1,422 lncRNAs are excluded as their repeat content rendered them unmappable, even in mouse.The relative expression of both coding and non‐coding clusters in each cell type.

The production of high‐quality gametes is essential to the propagation of life and the long‐term health of a species. Thus, cells of the germline and the molecular processes occurring within them carry special importance to the evolution of life. Spermatogenesis (Fig 1A) is a developmental process that ensures continuous production of spermatozoa and fertility in adult life 1. Spermatogenesis can be simplified to three distinct stages: mitotic, meiotic and spermiogenic. The mitotic component comprises spermatogonial populations containing spermatogonial stem cells and differentiating spermatogonia 2. These divide numerous times to amplify the pool of cells that will complete spermatogenesis, ensuring the production of large quantities of sperm 1. Thereafter, cells enter the meiotic phase undergoing DNA replication, chromosome recombination followed by two rounds of segregation generating haploid round spermatids. These subsequently enter terminal differentiation of spermiogenesis, converting these cells of round morphology into highly specialized spermatozoa 3. The processes of meiosis and spermiogenesis are associated with dramatic changes to the chromatin template and transcription itself (Fig 1A). Leptotene and zygotene (early stages of meiosis) are transcriptionally inert. Transition to pachytene coincides with resumption of transcription and genomewide loss of euchromatic repressive markers (H3K9me2) 4, 5. Furthermore, the fundamental nature of chromatin dramatically changes through spermiogenesis repackaging and compacting the haploid genome 3. This is achieved through successive replacement of the majority of histones with transitional proteins and then protamines 6. Previous studies clearly indicate that testes, at the whole‐tissue level, express a significantly greater number of transcripts than other tissues with particularly high long non‐coding (lncRNA) expression 7, 8, 9, 10. Furthermore, recently, systematic efforts have been made to understand the intricacies of changes in both transcription and the chromatin state throughout this developmental process through the analysis of specific cell populations 9, 11. Amongst other observations, these have defined a progressive transition to a permissive transcriptional state in post‐mitotic populations. This includes the general upregulation of a number of genomic elements, including several repeat classes 9. A unifying feature of all the listed chromatin alterations is that they expose the germline to the vulnerability of transposon mobilization via loss of these repressive markers.

Transposable elements (TEs) occupy a large fraction of mammalian genomes having colonized approximately 35–50% of human and mouse genomes 12, 13. In mouse, the two most significant classes of autonomous TEs are long interspersed nuclear elements (LINEs) and endogenous retroviruses (ERVs) occupying approximately 19 and 9% of the mouse genome, respectively 13. ERVs are retroviruses that colonized the germline and are then transmitted vertically across generations 14. Retroviruses code for a series of proteins (*gag, pro, pol and env*) flanked by two long terminal repeats (LTRs) that are essential for their replication 15. However, upon acquiring an endogenous lifecycle, the *env* protein is no longer required and may be lost, while the replicative components of ERVs retain the hardware required to copy‐paste themselves to novel locations 16. ERVs replicate via intermediate RNA genomes, with reverse transcription converting these to DNA for integration. The repetitive nature of the LTRs allows for effective replication of the viral ends 17. In addition, LTRs contain transcription factor binding sites (TFBS), a promoter and a polyadenylation signal 15, 18, 19. Due to the new set of selective pressures associated with vertical rather than horizontal transmission, not all ERVs remain complete 20. Copies often accumulate mutations or become fragmented over time 21, and frequently, solitary LTRs remain at an integration site following recombination between adjacent LTR regions 22.

Non‐LTR retrotransposons, such as LINEs and SINEs, are also prevalent 23 in vertebrate genomes (e.g. LINE1), and such elements are again capable of disrupting proximal gene activity either via specific internal features or through insertional mutagenesis 24. Thus, ERVs, LINEs and SINEs have the potential to be directly and highly mutagenic both in terms of insertional gene disruption and through gene deregulation associated with integration of their powerful regulatory elements. They also indirectly provide homology for non‐allelic recombination causing genomic deletions, inversions and duplications 24, 25. Thus, transposons have had major impact on the architecture, function and evolution of animal genomes.

Due to the features associated with their LTRs, ERVs are increasingly seen as both drivers of genome architecture but also as active players in shaping transcriptomes in tissue‐specific manners. ERVs have been shown to act as enhancers 26, 27, alternative promoters 28, splice sites with associated exonic sequences 29 and polyadenylation sites 18. LTRs have been observed as alternative promoters of individual protein‐coding genes. For example, the species‐specific insertion of LTRs regulate the NAIP locus 28 and in mouse an LTR acts as an alternative promoter for Dicer 30. High‐throughput sequencing increasingly illustrates the role of ERVs in genomewide transcription 31, 32. This has been most comprehensively demonstrated in embryonic tissues and pluripotent cells 33 where ERV‐associated transcripts have spliced into adjacent genes or genomic regions 34, 35. In the case of the mouse embryo, MuERV‐L enhancer co‐option drives the expression of over a hundred totipotency‐related genes at the two‐cell stage 35. However, ERVs have also been shown to have wider roles in other tissues such as the placenta where ERV *env* genes play an essential function in placental development and ERVs are enriched within enhancers, contributing transcription factor binding sites 27, 36. Finally, there is evidence that ERVs impact the female germline where ERV‐derived transcriptional start sites (TSSs) are a significant phenomenon 37 and the male germline where RLTR10B is linked as a promoter to at least 10 transcripts in testes 32.

lncRNAs have previously been postulated as a possible pool for deriving novel functionality and novel peptides 38, 39 Their rapid birth and death makes them a suitable substrate for this type of evolution 8, 10, 40, 41. In the testis, pervasive transcription has been hypothesized to underpin the emergence of novel transcripts 42. Hence, the germline represents a testing ground for transcriptional exploration and evolution, as one expects generally toxic and deleterious products to be rapidly eliminated.

Previous studies of the male germline reveal a highly complex and global RNA regulatory network of mRNAs, lncRNAs and piRNAs with TEs and pseudogenes acting as regulatory sequences 11, 43. Other studies clearly indicate that testes express a significantly greater number of transcripts than other tissues 8, 9. TEs have previously been found as functional domains within lncRNAs and have contributed to their origin, diversification and regulation 44, 45, 46. Profound changes in chromatin during spermatogenesis provide a window of opportunity for transposon activity, coupled with pervasive transcription of lncRNAs 8, thus creating a unique environment for transcript evolution. Hence, we sought to determine whether transposable elements drive this pervasive lncRNA transcription and contribute to *de novo* transcript genesis.

## Results

### Defining mouse spermatogenic transcriptomes

To explore features of the spermatogenic transcriptomes, we generated ribo‐depleted strand‐specific RNA‐Seq libraries isolated from several purified populations of mouse germ cells. The three principal stages of adult spermatogenesis were represented by *in vitro*‐cultured spermatogonial stem cell lines, meiotic spermatocytes and the haploid round spermatids (Fig 1A). In addition, erythroblasts (EBs) were used as a non‐germline out‐group for comparison. Samples were sequenced to high depth and reads were assembled *de novo* and *ab initio* and merged to produce a unified transcript set (Appendix Fig S1A). These transcripts were filtered, on splicing, length and cross‐assembly representation (Appendix Fig S1B and C) and grouped into transcriptional clusters sharing overlapping exons. Finally, the coding potential of all transcripts was determined using BLAST 47 and phyloCSF 48. Thresholds for determining the coding potential of loci were defined through comprehensive analysis of scores associated with known protein‐coding and non‐coding loci derived from Ensembl 49 (v69) (Appendix Fig S1D and E). In total, 68990 transcripts remained after filtering, representing 17058 clusters (Fig 1B). Of these clusters, 12,013 are protein‐coding, 3,424 were confidently ascribed as lncRNAs and 1,621 as “intermediate”, having only passed one of the coding potential tests (Fig 1B). The vast majority (92.6%) of clusters that overlapped a pseudogene loci were placed in the coding or intermediate classes and pseudogene‐associated clusters were depleted from the lncRNAs (Appendix Fig S2); 6,511 transcripts have not been previously identified in Ensembl (*v81*), and more than 75% of the lncRNA transcripts are novel (Appendix Table S1). The largest proportion of lncRNAs are classified as intergenic followed closely by those overlapping the 5′ TSS of known protein‐coding genes (Fig 1B).

To investigate the quality of assigned TSSs in our assembly, in the absence of cell type‐specific 5′ Cap analysis gene expression (CAGE) tags, we explored the 5′ localization of ENCODE 50 testis RNA Polymerase II (Pol II) ChIP‐Seq signals. As expected, coding transcripts have a strong TSS‐associated Pol II peak (Fig 1C). LncRNA transcripts also associate with Pol II peaks with a weaker signal, perhaps due to the lower level of expression of lncRNAs in general 7. To explore the quality of TSSs, we compared 5′ ends of assembled transcripts to FANTOM5 CAGE peaks (Appendix Fig S3A) and Ensembl annotation (Appendix Fig S3B). In general, TSSs associated with higher read depth more closely resemble those in Ensembl (Appendix Fig S3B). It is clear from these analyses that although a large number of our assembled TSSs have CAGE evidence, a proportion of the assembly likely represent fragments of transcripts. This is to be expected, as cell‐specific matched CAGE samples were not available for filtering (Appendix Supplementary Methods). However, even had these data been available, transcripts with repetitive promoter regions (such as those discussed below) would be discarded at a higher frequency when using approaches reliant on the mapping of tags to TSSs.

We inspected exon conservation across the assembly. Protein‐coding transcripts exhibit higher sequence conservation across their exons and in particular at splice junctions (Fig 1C), while lncRNA exons are far less conserved 8, 51, 52, 53. We next explored gene‐level conservation via homologue detection from 15 whole genomes (Fig 1D). Interestingly, many lncRNAs (42%) were not mappable to any species due to the presence of repetitive and low‐complexity sequences (median repeat and low‐complexity content 41% versus 9% in mapped, see Appendix Supplementary Methods). This homology‐based approach confirms the existence of sequence across species, but does not indicate however, whether such homologous regions are transcribed. Mappable lncRNAs exhibit low levels of conservation; 22% are unique to mouse and a further 41% are present only in mouse and rat. A small fraction (2.1%) are highly conserved and detectable in 10 or more species. Another striking facet of spermatogenic lncRNAs is that there is a dramatic increase in the lncRNA expression levels as spermatogenesis progresses (Fig 1E and Appendix Fig 4SB). At the round spermatid stage, lncRNAs expression approaches that of coding transcripts, at least in terms of log_10_(FPKM) values, although in raw expression terms, lncRNAs are still expressed at a lower level. This is in stark contrast to erythroblast controls, where lncRNAs expression is significantly lower than coding genes, in agreement with previously published data 7. Additionally, we observe that these highly expressed post‐mitotic lncRNAs are also more likely to be intergenic than any other class (Appendix Fig S4A). In summary, the post‐mitotic spermatocyte and round spermatid transcriptomes are characterized by a high frequency of abundantly expressed, clade‐specific and intergenic lncRNAs.

### The expression of ERV‐associated lncRNAs is a characteristic of post‐mitotic spermatogenic transcriptomes

We sought to determine the genomic origins of this class of upregulated spermatogenic lncRNAs. Spermatogenesis coincides with dramatic chromatin remodelling associated with derepression of certain TEs, although transposition is suppressed at the post‐transcriptional level 4, 54. Furthermore, TEs have been observed to be a mobile source of transcription factor binding sites and promoters 19, 28.

**Figure 2:**
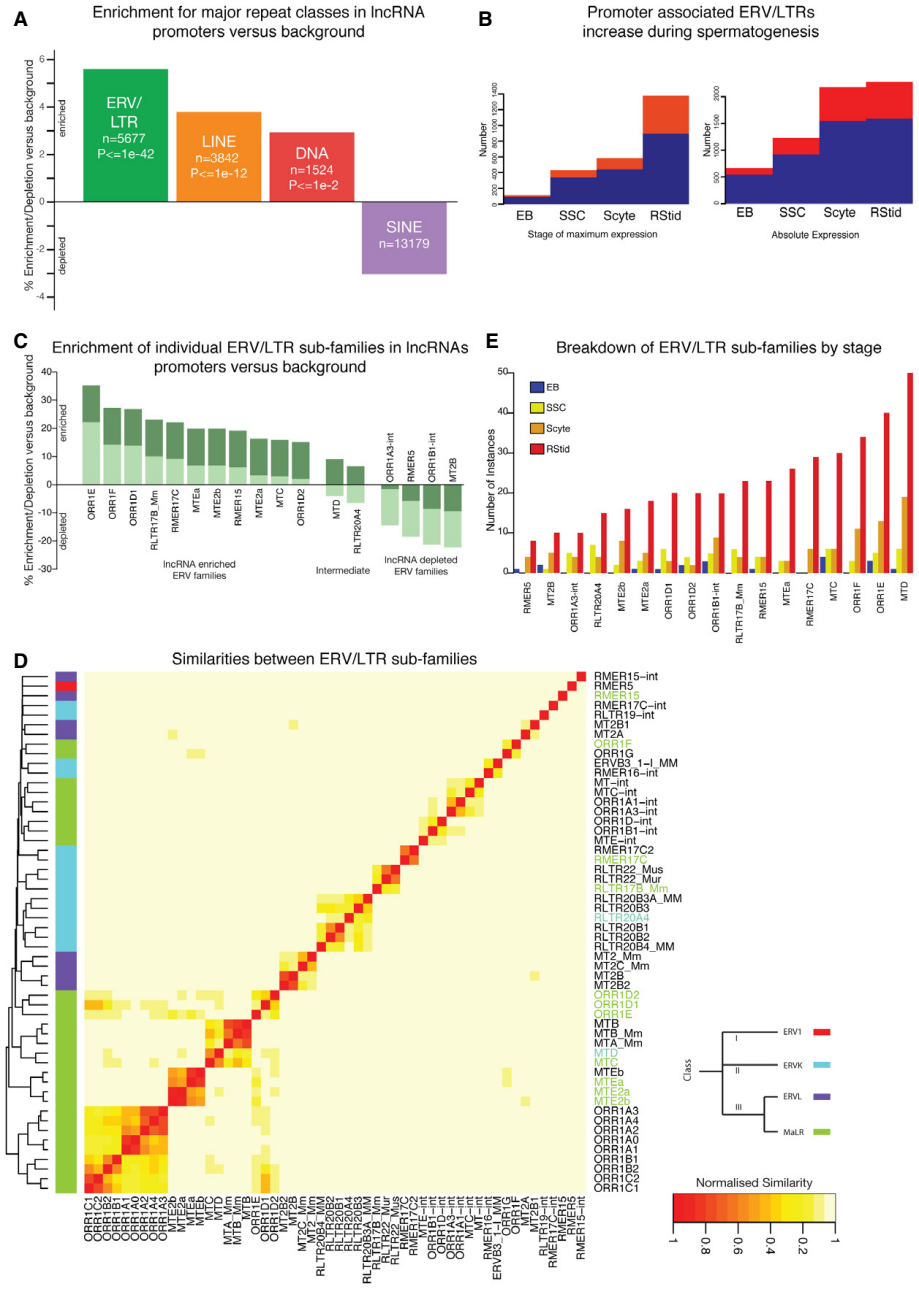
Increasing influence of transposable elements during spermatogenesis The shift in the proportion lncRNAs promoters associated with specific repeat classes relative to a genomewide background. Relative enrichments in mouse round spermatids for the four major classes are shown. Repeat classes are ordered according to adjusted *P*‐values (Holm's method) of enrichment, while “*n*” denotes the size of each promoter set. Only repeats with > 500 associated regions are shown.For each sample, the number of lncRNA clusters without (dark blue) and with (red) ERV/LTR elements in their promoter are shown. Left: Each lncRNA is assigned to the single sample where it had maximal expression. Right: Each lncRNA is assigned to any sample where its expression was above a minimum FPKM threshold. In both representations, only lncRNA clusters with a single TSS were used.Enrichments for lncRNA‐associated ERV/LTR subfamilies. Enrichment is computed based on the relative proportion (coding vs. non‐coding) of promoters associated with LTR elements. Individual bars represent relative non‐coding enrichment for specific ERV subfamilies. A conservative approach (light green bars) computes enrichment for non‐coding promoters relative to the non‐coding fraction of genomewide promoters that contain any ERV. A less conservative enrichment (dark green bars) is computed relative to the non‐coding fraction of promoters without any detectable ERV. Promoter sets where both conservative and less conservative enrichments are above zero are termed “lncRNA‐associated”. Those below zero for both measures are assigned as “background”. All other ERV subfamilies are termed “intermediate”.Heatmap of relationships between different ERV subfamilies bi‐clustered according to sequence similarity correlation. The colour key to the right of the plot corresponds to each element's ERV family.The number of non‐coding clusters with one or more promoter containing a relevant ERV element according to the cell type. Clusters are assigned to the cell type within which they exhibit their maximum expression.

We hypothesized that certain TEs have overcome suppression in the murine male germline to act as drivers of adjacent non‐coding transcription. If this were the case, one would expect a higher fraction of non‐coding transcription occurring near these elements. To test for this enrichment, we compared the presence of TEs within the promoters of both coding and lncRNA transcripts (Fig 2A). A number of elements were shown to be significantly enriched in lncRNA versus protein‐coding promoters. Of these, LTR elements were the most significant (*P* < 1 × 10^−42^). This relative increase in the proportion of non‐coding promoters associated with LTR elements is also reflected as a genomewide enrichment for the elements in the promoters and at the TSSs of lncRNAs expressed in round spermatids, particularly at higher expression thresholds (Appendix Table S2). Additionally, the observed increase in lncRNA expression in later stages of spermatogenesis is associated with an increasing fraction of LTR‐linked lncRNAs (Fig 2B). In round spermatids, up to 1,051 expressed lncRNA clusters (33%) are associated with one or more ERV overlapping either a promoter region or TSS (Appendix Fig S5). Interestingly, SINE elements and DNA elements are also enriched in lncRNA promoters (but, with the exception of SINE elements in EBs, not at the TSSs, Appendix Table S2). In the case of SINE elements, this enrichment is not reflected as a shift in the proportion of the associated promoters that are non‐coding (Fig 2A) and may represent a general enrichment of SINE elements in genic regions 55.

We next sought to understand whether this observed enrichment within lncRNA promoters is generic to all LTR elements or specific ERV subfamilies. Promoters containing ERVs were examined to identify those more likely to be associated with lncRNAs transcripts than expected. This analysis reveals a set of lncRNA‐associated ERV subfamilies enriched in proximal lncRNA transcripts relative to protein‐coding promoters (Figs 2C and E, and Appendix Fig S4C). Indeed, ORR1E is associated with the promoters or TSSs of 62 lncRNAs and only 85 protein‐coding genes. By contrast, MT2B is paired with 18 and 233 genes, respectively (Appendix Fig S6). The subfamilies most associated with lncRNA expression are members of class II & III ERVs 56. This set comprises: ORR1 (Class III), MT (Class III), RMER (Class II & Class III) and RLTR (Class II). Indeed, several related subfamilies of ORR1 (especially ORR1E) are linked to lncRNA expression (Fig 2D). Irrespective of the ERV subfamily classification, associated lncRNAs are upregulated at later stages of spermatogenesis (Fig 2E) with intergenic lncRNAs tending to make up a large fraction (Appendix Fig S4D). These associations are again reflected in ORR1E, RMER17C and MTE2b showing a genomewide enrichment in the promoters and at the TSSs of lncRNA transcripts, particularly in post‐mitotic cells (Appendix Table S2).

In summary, within the post‐mitotic spermatogenic transcriptome, many ERV subfamilies are highly associated with promoters of abundantly expressed lncRNAs.

### Select ERV elements act as lncRNA promoters in spermatocyte and round spermatids

**Figure 3:**
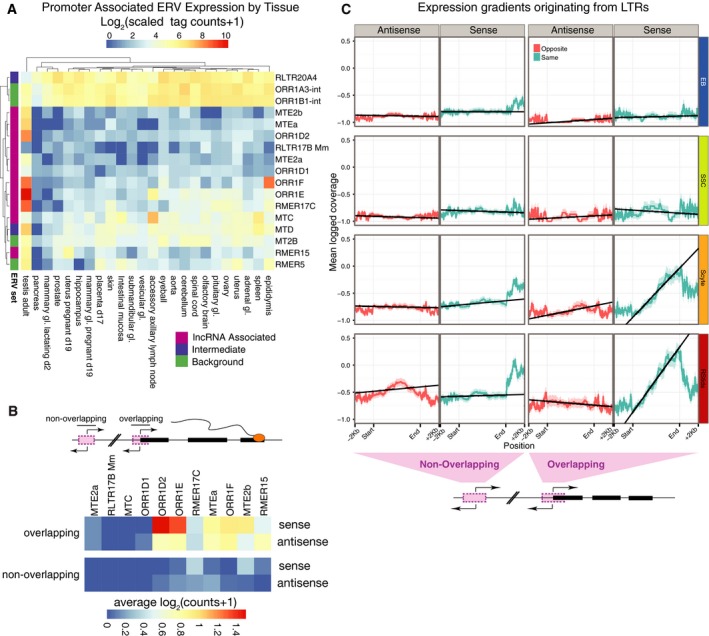
Analysis of ERV expression The CAGE expression (log2 scaled) of promoter‐associated ERV elements from selected subfamilies. When CAGE tags map multiple loci, these are split between repeats to avoid double counting. The colour bar (left) corresponds to the ERV set to which the ERV element belongs: either lncRNA‐associated, intermediate or background.The CAGE‐derived expression of lncRNA promoter‐associated ERV element sets found in the promoters of non‐coding transcripts, divided according to the relationship of the ERV to the closest adjacent TSS (overlapping or non‐overlapping, according to inset cartoon) and the orientation of the CAGE tags relative to the TSS (sense or antisense). Only uniquely mapped reads are considered.Mean cell type‐specific RNA‐Seq read coverage gradient across promoter‐associated ORR1E‐LTRs and their flanking regions for repeats overlapping a transcript TSS and those elsewhere in the promoter region. Coverage is reported in the sense and antisense orientation relative to the TSS. Each row depicts a representative replicate from one of the four cell types.

The above association between ERV elements and lncRNAs may indicate that LTRs of ERVs act as the actual promoters of these transcripts. Previous studies have also indicated this likelihood in somatic tissues 46. We sought to exclude the alternative possibilities that the observed effect is simply a bias for the insertion of subsequently silent ERVs into open chromatin proximal to active genes or that the ERV enrichment is a consequence of difficulties in read mapping and assembly across repetitive regions. To this end, we sought independent, transcriptome‐wide evidence to test for ERV promoter activity. We mapped a panel of FANTOM5 57 CAGE tags to the complete set of individual ERV elements. Those ERV elements found in the promoters of our assembly are indeed transcriptionally active in adult testes (Fig 3A). Although some other individual elements from the same subfamilies possess broader transcriptional profiles.

Next, we aimed to determine whether ERVs are actively driving lncRNA transcription. Here, we harness the strand specificity of CAGE tags. If non‐coding transcription was being driven by ERV elements, one would expect to observe CAGE‐derived strand‐specific expression associated with ERV elements to overlap TSSs. Indeed, this is precisely what is observed (Fig 3B, “overlapping”) with ERVs overlapping TSSs exhibiting strand‐specific (sense) expression. We also consider ERV elements not directly overlapping TSSs (Fig 3B, “non‐overlapping”). Strikingly, the expression of ERV elements present in the promoter (1kb) but not directly overlapping the TSS is considerably lower. In general, these elements remain relatively transcriptionally silent. Interestingly, when we compute similar data for coding transcripts (Appendix Fig S7), we also observe an effect, again only for overlapping ERV elements. However, here the effect appears to be predominantly antisense for many subfamilies of ERV. Hence, using FANTOM5 CAGE data, we confirm that ERV elements are a source of genomic transcripts in the testes.

As a complementary approach, we reanalysed our RNA‐Seq data using a method to allow directional TSSs to be easily recognized and to demonstrate the potential for transcriptional activity within repeat subsets. We divide promoter ERV elements into two sets according to whether they directly overlap the TSS of an assembled transcript regardless of coding potential. For each of these two sets, we compute the coverage of uniquely mapped reads across individual elements and then calculate the mean coverage across all aligned subfamily members. ERV sets enriched in active promoters are expected to produce a directional coverage gradient of RNA‐Seq signal, due to transcription starting at a TSS within each ERV and continuing in 3′ direction. On the other hand, ERV sets not acting as promoters are expected to have uniform or no RNA‐Seq coverage if they are part of longer transcripts or are not transcribed. Using the ORR1E element as a representative example (Fig 3C and Appendix Fig S8A), we observe a strong, sense coverage gradient in the 3′ direction of downstream transcripts across the TSS. In stark contrast, there is no directional antisense signal. This implies mono‐directional transcription beginning within the ERV element. Additionally, no signal in either direction is identified amongst ORR1E elements not overlapping an assembly TSS. Furthermore, this transcriptional gradient is observed only in late stages of spermatogenesis (Fig 3C and Appendix Fig S8A). Next, we assess such coverage gradients across all promoter‐associated ERV elements (Appendix Fig S8B) from lncRNA‐associated, background and intermediate subfamilies (Fig 2C). This analysis clearly demonstrates that lncRNA‐associated ERV elements exhibit similar expression patterns to the ORR1E LTR described above. The expression of intermediate and background ERV class members (Fig 2C) is again mono‐directional. However, it is no longer as restricted to meiotic and post‐meiotic stages. In conclusion, LTRs of several specific ERV subfamilies are active promoters driving lncRNA expression in late spermatogenesis.

### TE‐driven transcription is a conserved feature of vertebrate spermatogenic transcriptomes

**Figure 4:**
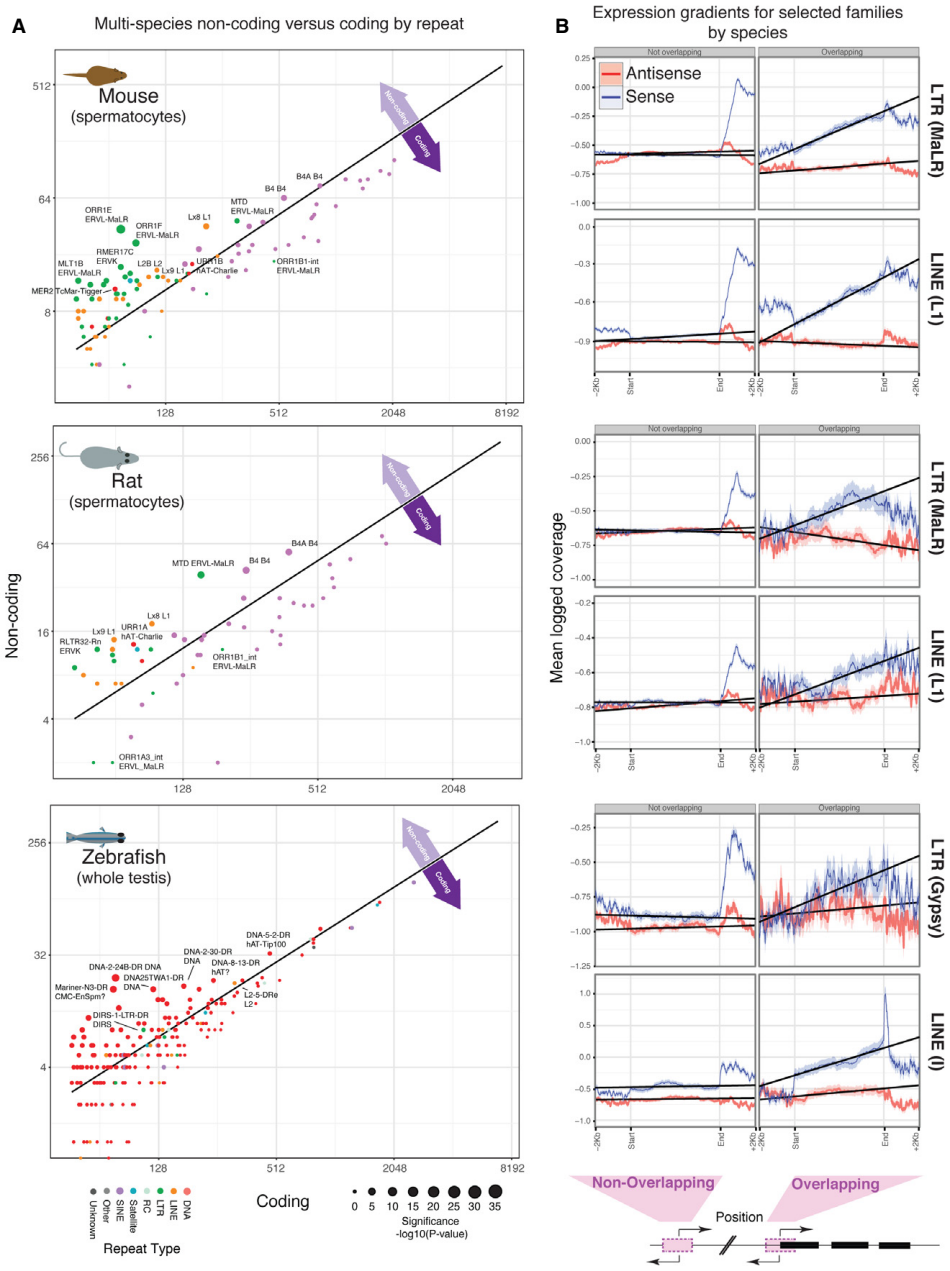
Germline transposon activity in three species Relative numbers of non‐coding (*y*‐axis) and coding (*x*‐axis) promoter regions containing specific repeat subfamilies for mouse (top), rat (middle) and zebrafish (bottom). The straight black line in all cases represents the expected levels based on the genomewide frequency of expressed promoter regions. Selected repeats are highlighted to illustrate non‐coding or coding‐enriched repeat families. Repeat classes and enrichment significance are indicated (inset legend). *P*‐values measure the significance of non‐coding promoter enrichment according to a hypergeometric test.The accompanying RNA‐Seq read coverage gradient plots for the most highly enriched non‐coding promoter‐associated repeat family from the LINE and LTR classes, in each of the three species. Coverage across repeat sets is provided in both sense and antisense orientations relative to the adjacent TSS for repeats either containing a TSS (overlapping) or which coincide with a 1‐kb promoter region (non‐overlapping). Coverage plots are provided for a single representative replicate.

Having observed ERV families driving expression of lncRNAs in spermatocytes and spermatids, we sought to determine whether this is a conserved feature of vertebrate spermatogenesis. We obtained and analysed data from rat (*R. norvegicus*) and zebrafish (*D. rerio*). Rat diverged from mouse approximately 30 Mya 58, while zebrafish diverged from mammals over 400 Mya. We performed strand‐specific RNA‐Seq from ribosomal‐depleted RNA isolated from rat spermatocytes and zebrafish whole testis. We tested these samples to determine whether a broad panel of promoter‐associated repeat elements are more likely to be associated with lncRNAs or coding genes. Again, we observe ERV elements as the most highly enriched repeats in lncRNA promoters in rat (Fig 4A and Appendix Fig S8C). We also observe LINE and SINE B4 elements enriched in both rat and mouse. The association of ERVs and LINE element families in mouse (promoter and TSS) and in rat (promoter) lncRNAs was confirmed by genomewide enrichments in these regions (Appendix Table S2). In contrast, zebrafish show abundant enrichment of DNA repeats for lncRNA promoters relative to their protein‐coding counterparts (Fig 4A) and LINE/LTR families are not significantly enriched in lncRNA promoters genomewide (Appendix Table S2). For all species considered, the vast majority of SINE element subfamilies showed no enrichment in lncRNA promoters and are skewed towards coding promoters (Fig 4A). Given these results and in the light of our initial enrichment analysis (Fig 2A), coverage gradient analysis was performed for the most significantly enriched family of repeats, from the LINE, LTR and DNA classes, in each species. We again sought to confirm that repeats are actively driving the expression of adjacent or overlapping TSSs (Fig 4B and Appendix Fig S8D–F). The MALR‐LTR elements drive expression of overlapping TSSs in both mouse and rat spermatocytes. However, zebrafish exhibits only a weak signal for Gypsy LTR‐driven expression in zebrafish testes. This may be a consequence of the use of whole testes samples or may suggest that LTR‐driven transcription is not as significant a component of germline transcription in teleosts. In contrast, LINE elements represent active promoter elements in all three species. DNA elements appear to be broadly transcriptionally silent (Appendix Fig S8D–F) with gradients much more difficult to discern, although there is some evidence that hAT‐Charlie repeats are transcriptionally active in rat spermatocytes (Appendix Fig S8E). In summary, LTR‐associated lncRNAs are a conserved feature of rodent meiotic transcriptomes; however, the phenomenon of repeat‐driven transcription can be expanded to include specific groups of LINE elements in mouse, rat and zebrafish.

### ERV‐derived lncRNAs as a source of transcript evolution

**Figure 5:**
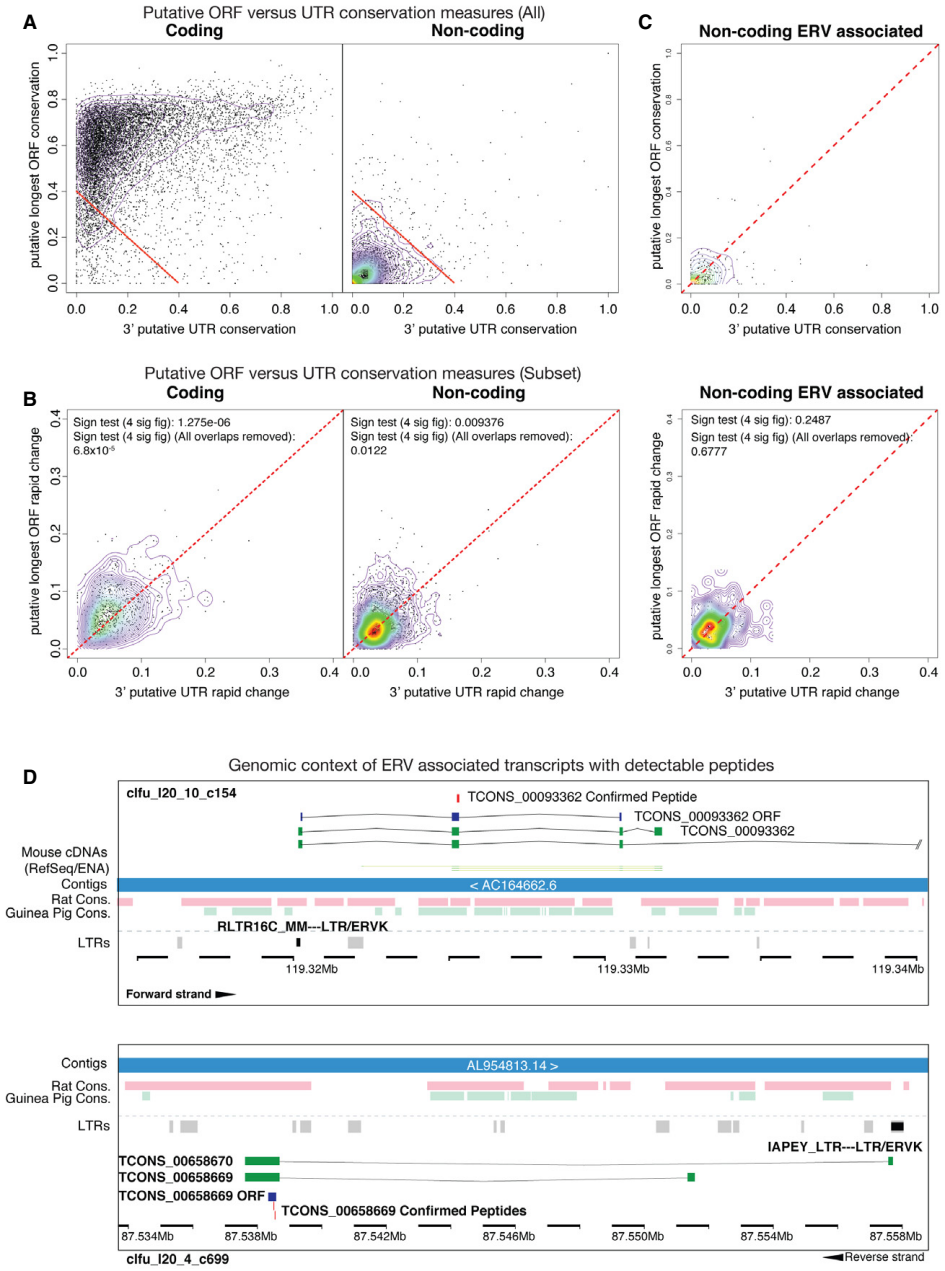
Transcripts annotated as non‐coding with putative open reading frames or detectable peptides The proportion of significantly conserved bases in the ORF versus paired 3′UTR of the longest ORF selected for each cluster for both coding (left) and non‐coding (right) loci. The red line represents the threshold for the selection of poorly conserved loci (sum of ORF and 3′UTR positive proportions < 0.4).The proportion of bases evolving at a significant rate in the longest ORF and paired 3′UTR at poorly conserved coding and non‐coding loci selected from (A). *P*‐values compare ORF proportions to those of the 3′UTR, according to a sign test.Relative conservation of the longest ORF from transcripts with an ERV element overlapping the TSS which were selected as representatives of non‐coding clusters. Top: Proportion of significantly conserved nucleotides. Bottom: Proportion of rapidly evolving nucleotides. In all cases contours are indicative of point density.Two examples of “non‐coding” clusters with ERV‐derived expression for which short peptides have been confirmed by mass spectroscopy. In each case, transcript models, cDNAs, confirmed peptides, longest ORF, associated repeat and other genomic features are indicated.

Having confirmed that ERV and transposable elements can drive the transcription of lncRNAs in vertebrate spermatogenic transcriptomes, we hypothesized that the expression of novel transcripts through the co‐option of LTR‐derived promoters could provide an opportunity to evolve novel non‐coding and/or coding genes. In the case of coding genes, open reading frames (ORFs) would evolve that could be translated into peptides. These emerging nascent ORFs would be subject to positive selection as they test, acquire or refine novel functionality. To test this hypothesis, we identified the longest ORF in each assembled protein‐coding and non‐coding transcript cluster. Subsequently, for each ORF, we calculated the fraction of bases undergoing negative selection or rapidly accumulating changes in the ORF compared to its putative 3′UTR. As expected, ORFs of protein‐coding transcripts show a greater proportion of bases undergoing negative selection (Fig 5A) as compared to their 3′UTRs. For lncRNAs, neither their ORFs nor their 3′UTRs are under clear negative selection. However, such ORFs could be rapidly evolving. To test this, we extracted subsets of non‐conserved ORFs to explore in detail (see Appendix Supplementary Methods). We observed a small but significant shift, with a greater proportion of bases in these ORF sets evolving rapidly as compared to their 3′UTRs (*P* < 0.01 & *P* < 1.3 × 10^−6^, lncRNAs and protein‐coding, respectively, Fig 5B). As one might expect, non‐conserved rapidly evolving protein‐coding transcripts are enriched for “immune response”‐related genes and “sperm–egg recognition” proteins (Appendix Tables S3–S5). The similar shift for lncRNAs is surprising. This suggests that at least some lncRNAs contain nascent novel ORFs, accumulating changes more rapidly than expected, perhaps under selective pressure. Based on ORF v 3′UTR comparisons, ORFs from ERV‐associated lncRNAs are not conserved as expected, showing a very slight shift towards more rapid evolution (Fig 5C). To understand whether ERV‐driven lncRNAs encode peptides from novel ORFs, we subjected spermatocyte and round spermatid proteins to LC‐MS/MS mass spectrometry looking for such peptides. From 175 candidate ERV‐driven ORFs, we identified peptides corresponding to 23 of the selected ORFs (Fig 5D and Appendix Table S6) and further confirmed their existence with targeted mass spectrometry. Such peptides are unlikely to be functional, yet may serve as precursors to functional peptides via evolution.

### Activity of ERV elements as promoters in somatic tissue

**Figure 6:**
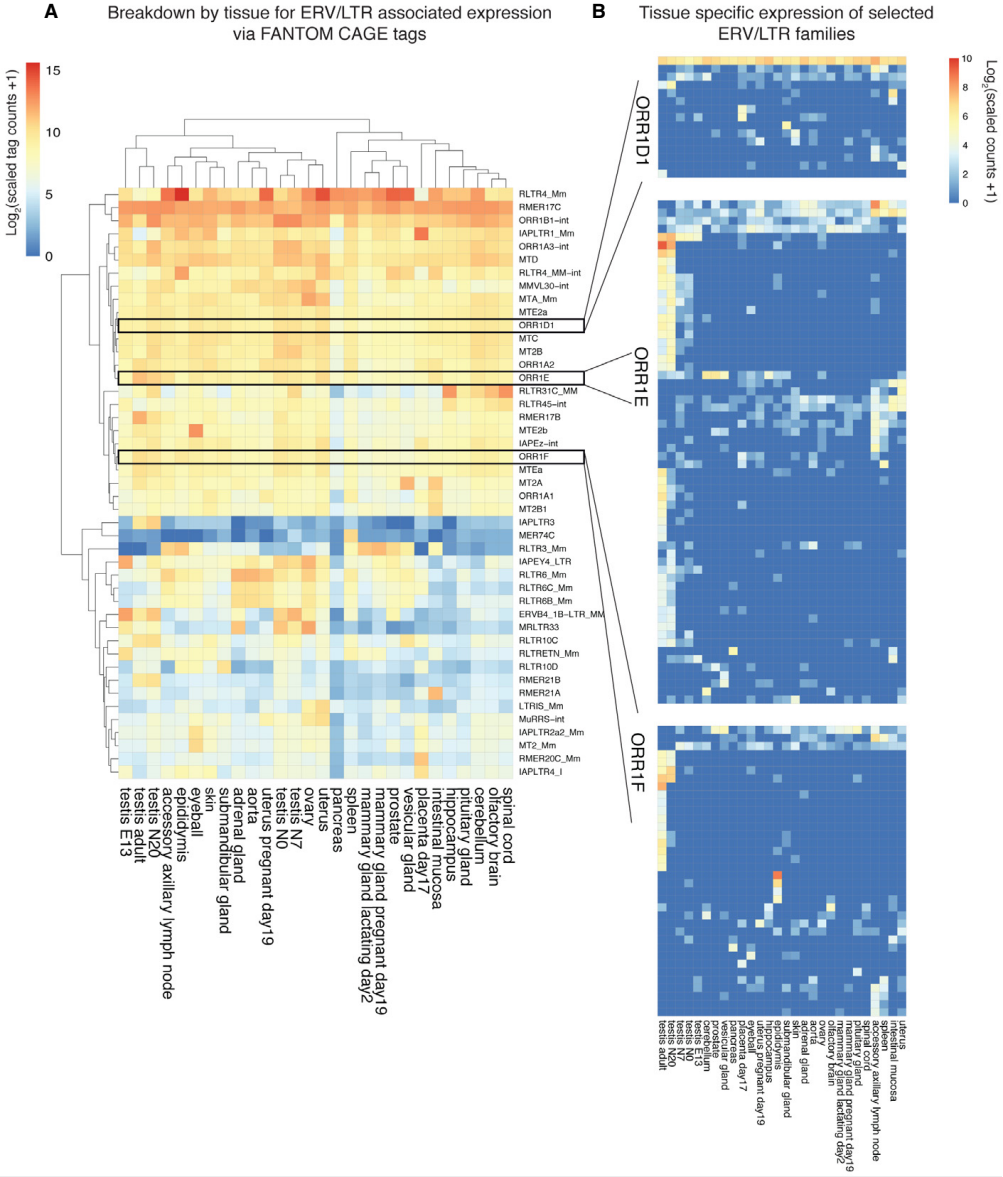
ERV transcription across multiple tissues by family and subfamily CAGE‐derived expression heatmap for ERVs in a panel of 28 tissues including somatic tissues. Tissues and ERV subfamilies are bi‐clustered according to their correlation. Tissues and repeat subfamilies with similar patterns will usually group together. The depth of multimapping reads are divided across repeats. A scaled depth of 1,000 in one or more tissue is required for inclusion.CAGE‐derived expression of individual repeat elements from the ORR1D1, ORR1E and ORR1F subfamilies. The depth corresponds to uniquely aligned CAGE tags with a scaled depth of 10 required in one or more tissue for inclusion.

Our results indicate ERVs are drivers of major transcriptional plasticity in the male germline. Previously, Faulkner *et al* 31 performed a thorough analysis of retrotransposon‐derived TSSs using FANTOM 4 CAGE data. We therefore expanded the FANTOM5 CAGE analysis to explore the extent to which our ERV subfamilies of interest perform as active promoters in other tissues (Fig 6A). These data show distinct patterns of ERV expression both between tissues and across ERV subfamilies, confirming the results of the earlier work 31. However, given that the expansion in ERV‐driven lncRNA expression in the later stages of male gametogenesis, we were surprised to see that some of the ERV subfamilies associated with this process appear to be almost ubiquitously expressed across germline and somatic tissues. We therefore investigated the expression of individual repeats for three members of the ORR1 group of elements (Fig 6B). Remarkably, individual repeats within the same subfamily have very distinct expression profiles. This is perhaps most striking for ORR1E with many elements expressed almost exclusively in the male germline, while others are either expressed in many tissues or upregulated in the accessory axillary lymph node, spleen, intestinal mucosa and uterus.

Having established that ERV elements are expressed across many tissues, we wished to expand this analysis further to explore whether ERV‐regulated lncRNA expression is phenomenon‐restricted to the germline. Using Ensembl annotation and mouse ENCODE RNA‐Seq data, we identified tissue‐specific lncRNAs in a panel of 13 tissues. For these genes, we assessed the absolute number and fraction of transcripts with a promoter or TSS‐associated ERV element (Appendix Fig S9). From this assessment, it is clear that although LTR‐associated lncRNA transcription is most apparent in testes where more than 350 tissue‐specific lncRNA transcripts have an ERV element overlapping their TSS, a comparable fraction of tissue‐specific lncRNA TSSs overlap an ERV element in adipose tissue, lung and liver. At a more granular level, many of the subfamilies most frequently associated with testis‐specific lncRNA TSSs match those identified by our earlier analyses (Appendix Fig S10).

These results indicate that although the germline is a significant source of ERV‐driven transcription, ERVs can drive transcription across many tissues in a highly regulated manner.

## Discussion

We present one of the most comprehensive analyses to date of non‐coding transcription during spermatogenesis, complementing the work of previous studies 9, 11. We were able to achieve high developmental resolution by assembling the transcriptome from ribo‐depleted RNA‐Seq data sets derived from sorted populations representing the principal stages of spermatogenesis, and subsequently, we link this to repeat element expression. Specifically, we have made particular effort to perform comprehensive analyses of the quality of our transcriptome assembly and lncRNA models. Our analysis of repeat expression and its association with large numbers of lncRNAs in small cell populations highlights many issues hampering our understanding of these phenomena. It is clear from the large number of novel transcripts identified in this study (Appendix Table S1) that in order to fully understand the transcriptomes of specific cell types, transcriptome assembly is essential. However, in these situations, independent and matched end evidence is difficult to ascertain and in many cases challenging to map to a single specific loci. We believe that our data set can also provide a backbone for further improvements and observations. Importantly with respect to this work, we confirmed many of our observations using alternative approaches, incorporating data sets from Ensembl, ENCODE and FANTOM 49, 50, 57.

As previously described we note an increase in promiscuous transcription in spermatocytes and round spermatids 9. However, uniquely, we observe MaLR, ERVK and ERVL ERVs driving significant expression of lncRNAs in mouse post‐mitotic spermatocyte and round spermatid populations, with the ORR1 family the most striking example. lncRNA expression is characterized by dramatic increases in the number of loci expressed and their overall degree of expression as spermatogenesis progresses, peaking at the round spermatid stage. As the testis has the most pervasive expression of lncRNAs as compared to other organs 8, 9, our observation of LTR‐driven transcription in post‐mitotic spermatogenic cells may in a large part explain this phenomenon. TEs have been shown to be involved in the shaping of lncRNA functional domains 45 and it is now widely accepted that they can form active endogenous promoters, driving the expression of sets of lncRNAs, in particular in embryonic stem cells and the early embryo. However, here we show them actively driving the transcription of significant wave of lncRNAs in the post‐mitotic male germline. Hence, these elements act as an origin for the expression of species‐ and clade‐specific genes. As such, these findings further corroborate an observation from the mouse ENCODE project which found ERV1, ERVK, ERVL and MaLR subfamilies to be enriched in mouse‐specific promoters 59. ORR1E elements appear to be particularly active in driving lncRNA expression. This is relevant in the light of previous work that demonstrated that in rodents, the predominant promoter for *Naip* genes is an ORR1E element, in contrast to the human copy of the gene 28. This suggests ORR1E may play a particularly important role in the derivation of novel promoters. However, although ERVs represent the repeats most highly enriched in lncRNA promoters, they are far from alone in this association with lncRNAs, with LINEs also clearly implicated.

The activity of transposable elements is regulated by a number of mechanisms, and the onset of LTR‐driven lncRNA expression coincides with genomewide loss of the euchromatic H3K9me2 repression 4. H3K9me2 is known to be resident and repress LINE1 elements until the late zygotene stage 60. In addition to the upregulation of transcription factors that may bind respective LTRs, it could be that the genomewide loss of H3K9me2 unleashes this wave of ERV‐driven transcription. Indeed, CpG DNA methylation alone is insufficient to repress both IAP and LINE1 elements during meiosis 4.

The germline is conceptually an ideal location to select and evolve transcript function, as any broadly toxic gene product (RNA or protein) would be rapidly eliminated through deleterious effects on gametogenesis. This “out of the testis” hypothesis has been described previously by Kaessmann 42. To investigate further, the work by Soumillon *et al* 9 explored the increased transcription of duplicate genes and intronless retrocopies in this unique environment and also noted an increase in lncRNA transcription. It is this lncRNA transcription and its potential that we have aimed to describe in more detail. The presence of mobile and fully formed promoters endows the vertebrate genome with the opportunity to rapidly innovate genetic products. Although large numbers of these TEs would be silent in most somatic tissues, their activation in post‐mitotic spermatogenic cells provides an opportunity for the transcriptome to explore expansive genomic space for potential *de novo* gene genesis and subsequent selection of ORFs within lncRNA transcripts. By connecting ERV promoters presented here to extensive annotation of associated lncRNAs, we were able to investigate whether these transcripts could be further selected for function. As a first step, we have begun to explore their potential for the evolution of protein‐coding loci. Genesis of functional protein‐coding loci from intergenic regions and lncRNA has been demonstrated 35, 38, 39, 61 and in some cases linked to the testis 62, 63. However, there remain many unanswered questions regarding the mechanisms involved in these processes. Our identification of peptides from a subset of ERV‐driven lncRNAs suggests TE promoters could facilitate the male germline as a source of protogenes 61. Given that most lncRNAs are species‐specific or restricted to a closely related clade 64, the mechanisms described here may have special importance in *de novo* genesis of both lncRNAs and peptide‐encoding loci.

Intriguingly, our analysis of FANTOM5 CAGE data demonstrated that LTR‐driven transcription can be far more widespread than we were expecting. This is in agreement with the work of Faulkner *et al* 31 where the authors went further to demonstrate, as an example, the tissue specificity of individual members of the VL30 subfamily of LTRs using an earlier iteration of the FANTOM data. Subsequent to this earlier analysis, we were able to investigate the expression of repetitive elements to a relatively high resolution using the latest CAGE data. In doing so, it appears that individual LTRs of subfamilies selected as drivers of lncRNA expression in gametogenesis themselves have intricate, divergent and tissue‐specific expression profiles (Fig 6). Similarly intricate cell‐specific expression patterns have been noted in the early embryo 34. This is complemented by our findings when using Ensembl annotation to define promoter regions and ENCODE sequence data (Appendix Fig S10). Although the extent to which LTRs influence expression in testes appears to be relatively unparalleled (Appendix Fig S9), the phenomenon itself is ubiquitous with the potential for much broader impact, extending beyond the germline adding to the expanding literature describing similar phenomena.

Finally, we can show that retrotransposon‐driven expression is a conserved feature of vertebrate spermatogenesis and plays a particularly significant role in driving and regulating lncRNA expression in rodents. Understanding the extent to which ERVs rewire transcriptional networks will be an important future direction, but this work helps to transfer ERVs from their traditional status as parasitic and opportunistic DNA elements to promoter elements with a major influence on the regulation, diversification and evolution of vertebrate transcriptomes.

## Materials and Methods

For full detail of experimental methods in all cases, please refer to the Appendix.

### Sample preparation and sequencing

Per each cell type and species, libraries were prepared from two biological replicates. Erythroblasts were differentiated from E12.5 mouse livers *in vitro* 65, 66. SSCs were cultured *in vitro* as described previously 67. *Ex vivo* germ cells were obtained from dissection of 8‐week‐old adult mice through enzymatic digestion and mechanical disaggregation in EKRB buffer as previously described 68. Mouse spermatocytes and round spermatids were isolated and purified through Becton Dickinson Aria II cell sorter upon staining with Hoechst DNA dye as previously published 69. Adult rat spermatocytes were obtained and purified through a similar procedure. Zebrafish were raised and maintained using standard procedures 70. Whole testes were dissected from 1‐year‐old adult male AB zebrafish. Mice used were inbreed C57BL/6N strain, rats were Sprague Dawley^®^ strain purchased from Charles River. Mice were maintained at the EMBL Mouse Biology Unit, Monterotondo, in accordance with Italian legislation (Art. 9, 27 January 1992, number 116) under licence from the Italian health ministry. RNA was prepared via Qiazol lysis followed by DNase treatment in the presence of an RNase inhibitor. RNA was recovered via EtOH precipitation; 5 μg of RNA was ribodepleted using Ribo‐Zero (Illumina), and 50 ng of this RNA was used for strand‐specific cDNA library preparation (ScriptSeq, Illumina). Libraries were purified and analysed on High‐Sensitivity DNA chip (Agilent) on BioAnalyzer, and each paired‐end library was sequenced on one lane of a HiSeq 2000 sequencer.

### Transcriptome assembly

Two biological replicates from erythroblasts, spermatogonial stem cells, spermatocytes and round spermatids were processed. Adapter contamination was removed, replicates merged and sequences de‐duplicated such that each sequence is unique per sample using Kraken 71. Transcriptomes were assembled using two approaches, Cufflinks and Trinity 72, 73. For Cufflinks, reads were mapped using TopHat2 73. Trinity transcripts were remapped to the genome using gmap 74. Cuffmerge merged these into a unified assembly. Transcripts were discarded if they matched any of these criteria: length < 200 nt, maps to supercontig, is unspliced or has no strand. Unspliced transcripts were removed to mitigate DNA contamination and remove retrocopies. The assembly was further refined by comparing the original eight assemblies to the unified assembly using a Jaccard score overlap test. Transcripts below a cumulative Jaccard threshold (< 2.5) were discarded. This threshold was based on CAGE and polyA data. Filtered assembly transcripts were clustered according to exonic overlap, generating a set of related, transcript clusters encoding multiple isoforms.

### Transcriptome classification and analysis

Transcripts were assigned as non‐coding if they had a PhyloCSF 48 score < 50 (based on 29 species UCSC alignments 75) and no BLASTx match (E > 1 × 10^−10^) versus Ensembl 49 peptides and PfamA/B 76. Transcripts were assigned as “intermediate” if they matched only one of those criteria. In general, expression was obtained using FPKM values from RSEM 77 and the replicate mean was used. For heatmap expression visualization, global comparisons of major repeat classes and the three‐species analysis, DESeq2 78 was used to normalize and transform raw counts generated by HTSeq 79. Transcript genomic classification was determined with respect to Ensembl annotations including external enhancer data. An overlap of 1nt is counted as a genomic feature match. Matches are assigned by strand or as “both” where an overlap is not stranded. Pseudogene annotation was also derived from Ensembl. For RNA Pol II analysis, ChIP‐Seq data from ENCODE 50 were used. Reads were merged between replicates and remapped to the genome (mm10, Bowtie2). Coverage was computed and visualized using Bedtools2 and deepTools 80, 81. When multiple isoforms have TSS sites within 50 bp, only the longest isoform is retained. For exon conservation analysis, exons of the longest isoform of each transcript were matched to PhyloP 82 scores for the mouse genome (UCSC). To assess the quality of TSS annotation, the assembly was compared to FANTOM5 CAGE peaks 57. Bedtools was used to identify the closest peak to the TSS of transcripts from coding and non‐coding clusters. These distances were compared to those of Ensembl transcripts. To assess TSS annotation relative to read depth, the expression of TSSs was measured using RSEM (v1.2.7) 77. TSSs from coding loci were divided by expression quantiles and the distance to the nearest Ensembl protein‐coding promoter was calculated. Non‐coding TSSs were separated using the same thresholds.

### Repeat and ERV analysis

Repeat annotation was obtained using RepeatMasker, NCBI/RMBLAST and the RepeatMasker database of elements (v20130422) for mouse, rat and zebrafish. When non‐overlapping repeat analysis is performed, the lowest scoring repeat match at overlapping sites is trimmed until no overlaps remain. Promoter analysis was performed by searching for overlaps between repeats and sites 1 kb upstream of defined TSSs. Unless otherwise stated (see Appendix Supplementary Methods), when non‐redundant promoter analysis is performed a random transcript from each transcript cluster is selected, the promoter is defined and all overlapping promoters are excluded. For ERV expression gradient analysis, aligned reads (Tophat2) were filtered to remove multimappers and retain only the first of each pair. deepTools was used to calculate positive and negative strand coverage of ERV elements overlapping promoter regions and TSSs and their flanking regions (± 2 kb). When assessing the impact of ERVs on the non‐coding transcriptome, promoter and TSSs overlapping ERVs were considered independently and clusters were assigned the TSS set in preference.

### Promoter enrichments

Zebrafish and rat annotation was downloaded from Ensembl (v81), filtered and assigned a coding potential (see Appendix Supplementary Methods). Gene expression was derived from HTSeq counts and genes were filtered on the specified threshold. For the remaining non‐coding genes, promoters (1 kb upstream of transcripts) and TSSs (± 200 nt) were determined. Within each set, regions with a non‐strand‐specific overlap were merged. Effective genome sizes were calculated for each species. The least frequent repeat sets were removed from the analysis. Within each repeat set, overlapping repeats were merged and the central nucleotide considered as representative. A binomial test was used to assess an enrichment of repeats falling within the non‐strand‐specific, merged promoter regions. *P*‐values were adjusted according to the Hochberg method. For comparison of coding to non‐coding promoter proportions, briefly, repeats were divided according to family, subfamily or class. Overlapping repeats in each set were merged. Expression was determined as above, and for each cell type, a minimum threshold of 0.25 FPKM was used. Promoters with non‐strand‐specific overlaps were merged. These promoter regions representing solely coding or non‐coding genes were retained. Promoter regions overlapping the repeat sets were counted. Repeat sets with fewer than 50 representative promoter regions were discarded. A hypergeometric test was performed to find repeat sets with a non‐coding/coding promoter region enrichment. The mouse ENCODE tissue analysis was performed by mapping 13 paired‐end strand‐specific samples to the mouse genome followed by count quantitation, normalization and generation of FPKM values where necessary (see Appendix Supplementary Methods).

### Evolutionary analysis

Detection of conserved lncRNAs was performed via mapping the longest isoform from each lncRNA cluster against 15 genome sequences obtained from Ensembl using WU‐BLAST 83 seed matches followed by realignment as previously published 84. For longest ORF analyses, the longest complete ORF was selected for each transcript cluster. Conservation per nucleotide was calculated using PhyloP (mm10 genome, 60‐way vertebrate alignment, UCSC). For each transcript, the fraction of nucleotides with positive or negative PhyloP scores was computed separately (cut‐off = 1.301; *P* ≤ 0.05) for both the ORF and paired 3′UTR.

### CAGE analysis of repeat expression

Cap analysis gene expression tags were retrieved from FANTOM5 57. rRNA matches were filtered using *swan* (Kraken), and reads that map to a repeat masked genome were discarded (Bowtie, up to 3 mismatches). Remaining CAGE tags were mapped to a database of sequences for individual, non‐redundant ERV elements (Bowtie, 2 mismatches). Read depth was split between multimapped reads. Both uniquely mapped and multimapped counts were used.

### Mass spectrometry

A database for LC‐MS/MS search was created from a set of ranked ORFs selected based on a series of criteria (see Appendix Supplementary Methods). Spermatocyte and round spermatid cell extract preparation and digestion were performed 85. Peptides (200 ng) were injected on EasySpray 50 cm column (Thermo) connected to an Orbitrap Fusion Lumos (Thermo). Mascot (Matrix Biosciences) was used to search for matches with the following settings: MS1 tolerance: 5 ppm, MS2: 0.1Da, max missed cleavages: 2. Samples were re‐analysed with targeted MS, when only ERV‐derived peptides selected from previous experiment were measured. Targeted data extraction (MS1 filtering and PRM) was performed using Skyline 3.5 86 with tolerances of dotp > 0.75, idotp > 0.8.

### Data access

Primary sequencing data, assembled transcripts and ancillary data are available via the European Nucleotide Archive (Study: *PRJEB15333*) and the European Bioinformatics Institute (http://www.ebi.ac.uk/research/enright/testome).

## Author contributions

MPD led the analysis of the data and performed the core computational analyses. CC contributed to the design analysis and generation of the library/preparation of the samples. HKS contributed to the analysis and the generation of the assembly. SvD, TL, GB, JMM and AJE contributed to the analysis. TA performed the mass spectrometry under the guidance of RCA and JR. AS and AB contributed to the analysis and provided the zebrafish samples. AJE and DO conceived and supervised this study. MPD, AJE and DO wrote the final version of the manuscript.

## Conflict of interest

The authors declare that they have no conflict of interest.

## Supporting information

Appendix

Review Process File
