# Supplementary material for: Transposon‐driven transcription is a conserved feature of vertebrate spermatogenesis and transcript evolution
Source: EMBO Rep. 2017 May 12;18(7):1231–47. doi: 10.15252/embr.201744059 (PMC5494522; doi:10.15252/embr.201744059)
Supplement: Supplementary file 1 — Appendix [file EMBR-18-1231-s001.pdf]

Appendix: Transposon-driven transcription is a conserved feature of vertebrate spermatogenesis and transcript evolution

Davis *et al, 2017*

Table of Contents

Table of Contents	Page 1
Appendix Table 1	Page 2
Appendix Table 2	Page 2
Appendix Table 3	Page 8
Appendix Table 4	Page 8
Appendix Table 5	Page 9
Appendix Table 6	Page 9
Appendix Figure Legends	Page 10
Appendix Figure 1	Page 13
Appendix Figure 2	Page 14
Appendix Figure 3	Page 15
Appendix Figure 4	Page 16
Appendix Figure 5	Page 17
Appendix Figure 6	Page 18
Appendix Figure 7	Page 19
Appendix Figure 8	Page 20
Appendix Figure 9	Page 21
Appendix Figure 10	Page 22
Methods	Page 23
References	Page 37

Appendix Tables

Table S1

	Min. 1nt overlap	Min. 10% overlap	Min. 50% overlap
**non-coding transcripts matching Ensembl lncRNAs**	**1497**	**1383**	**807**
% of assembly non-coding transcripts	24.6%	22.7%	13.3%
**All assembly transcripts matching Ensembl annotation**	**62479**	**61261**	**50338**
% of assembly transcripts	90.6%	88.8%	73.0%

The assembly compared to Ensembl annotation. The comparison was performed for both transcripts from non-coding clusters against Ensembl lncRNAs and the assembly as a whole against all Ensembl transcripts. Percentages are with respect to the transcriptome assembly.

Table S2

Mouse: Repeat class enrichment

0.25 FPKM minimum cutoff: Non-coding promoter regions:

Promoter	number of repeats	eb	ssc	sc	rs
DNA_class	151415	1.0000	0.0936	0.0021	0.0004
LINE_class	832816	1.0000	1.0000	1.0000	1.0000
LTR_class	876647	1.0000	1.0000	1.0000	0.6300
Other_class	17857	1.0000	1.0000	1.0000	1.0000
Satellite_class	27996	1.0000	1.0000	1.0000	1.0000
SINE_class	1377369	0.0000	0.0000	0.0000	0.0013

TSSs	number of repeats	eb	ssc	sc	rs
DNA_class	151415	1.0000	1.0000	0.1908	0.4420
LINE_class	832816	1.0000	1.0000	1.0000	1.0000
LTR_class	876647	1.0000	1.0000	0.1848	0.0446
Other_class	17857	1.0000	0.6148	0.9782	1.0000
Satellite_class	27996	1.0000	1.0000	1.0000	1.0000
SINE_class	1377369	0.0022	1.0000	1.0000	1.0000

1 FPKM minimum cutoff: Non-coding promoter regions:

Promoter	number of repeats	eb	ssc	sc	rs
DNA_class	151415	1.0000	1.0000	0.0107	0.0004
LINE_class	832816	1.0000	1.0000	1.0000	1.0000
LTR_class	876647	1.0000	1.0000	1.0000	0.0099
Other_class	17857	1.0000	1.0000	1.0000	1.0000
Satellite_class	27996	1.0000	1.0000	1.0000	0.4573
SINE_class	1377369	0.0000	0.0000	0.0000	0.1104

TSSs	number of repeats	eb	ssc	sc	rs
DNA_class	151415	1.0000	1.0000	0.7457	0.6530
LINE_class	832816	1.0000	1.0000	1.0000	1.0000
LTR_class	876647	1.0000	1.0000	0.7641	0.0038
Other_class	17857	1.0000	1.0000	1.0000	1.0000
Satellite_class	27996	1.0000	1.0000	1.0000	0.9597
SINE_class	1377369	0.0001	0.4887	1.0000	1.0000

Mouse: Selected ERV sub-family enrichments

0.25 FPKM minimum cutoff: Non-coding promoter regions:

Promoter	number of repeats	eb	ssc	sc	rs
MT2B|ERVL	12124	0.9995	0.9890	0.8980	0.8342
MTC|ERVL-MaLR	26995	0.9995	0.9890	0.8980	0.8342
MTD|ERVL-MaLR	50330	0.9995	0.9890	0.8980	0.8342
MTE2a|ERVL-MaLR	15338	0.9995	0.9890	0.8980	0.8342
MTE2b|ERVL-MaLR	13628	0.9995	0.9890	0.8980	0.8342
MTEa|ERVL-MaLR	19293	0.9995	0.9890	0.8980	0.8342
ORR1A3-int|ERVL-MaLR	9537	0.9995	0.4642	0.3549	0.8342
ORR1B1-int|ERVL-MaLR	27711	0.9995	0.9890	0.8980	0.8342
ORR1D1|ERVL-MaLR	21490	0.9995	0.9890	0.8980	0.8342
ORR1D2|ERVL-MaLR	14702	0.9995	0.9890	0.8980	0.8342
ORR1E|ERVL-MaLR	21752	0.9995	0.9890	0.0156	0.0059
ORR1F|ERVL-MaLR	22291	0.9995	0.9890	0.8980	0.7434
RLTR17B_Mm|ERVK	28460	0.9995	0.9890	0.8980	0.8342
RLTR20A4|ERVK	21113	0.9995	0.9890	0.8980	0.8342
RMER15|ERVL	16421	0.9995	0.9890	0.8980	0.8342
RMER17C|ERVK	7233	0.9995	0.0000	0.0000	0.0000
**TSSs**	**number of repeats**	**eb**	**ssc**	**sc**	**rs**
MT2B|ERVL	12124	1.0000	0.9985	0.9992	1.0000
MTC|ERVL-MaLR	26995	1.0000	0.9985	0.9992	1.0000
MTD|ERVL-MaLR	50330	1.0000	0.9985	0.9992	1.0000
MTE2a|ERVL-MaLR	15338	1.0000	0.9985	0.9992	1.0000
MTE2b|ERVL-MaLR	13628	1.0000	0.9985	0.0455	0.0711
MTEa|ERVL-MaLR	19293	1.0000	0.3832	0.2218	0.5950
ORR1A3-int|ERVL-MaLR	9537	1.0000	0.9985	0.9992	1.0000
ORR1B1-int|ERVL-MaLR	27711	1.0000	0.9985	0.9992	1.0000
ORR1D1|ERVL-MaLR	21490	1.0000	0.9985	0.9992	1.0000
ORR1D2|ERVL-MaLR	14702	1.0000	0.9985	0.9992	1.0000
ORR1E|ERVL-MaLR	21752	1.0000	0.0060	0.0002	0.0000
ORR1F|ERVL-MaLR	22291	1.0000	0.9985	0.2218	0.0687
RLTR17B_Mm|ERVK	28460	1.0000	0.9985	0.9992	1.0000
RLTR20A4|ERVK	21113	1.0000	0.9985	0.9992	1.0000
RMER15|ERVL	16421	1.0000	0.9985	0.9992	1.0000
RMER17C|ERVK	7233	1.0000	0.0020	0.0000	0.0000

1 FPKM min cutoff: Non-coding promoter regions:

Promoters	number of repeats	eb	ssc	sc	rs
MT2B|ERVL	12124	1.0000	0.9441	0.9835	0.7793
MTC|ERVL-MaLR	26995	1.0000	0.9441	0.9835	0.7793
MTD|ERVL-MaLR	50330	1.0000	0.9441	0.9835	0.7793

MTE2a|ERVL-MaLR	15338	1.0000	0.9441	0.9835	0.7793
MTE2b|ERVL-MaLR	13628	1.0000	0.9441	0.9835	0.7793
MTEa|ERVL-MaLR	19293	1.0000	0.9441	0.9835	0.7793
ORR1A3-int|ERVL-MaLR	9537	0.1779	0.3833	0.9835	0.7793
ORR1B1-int|ERVL-MaLR	27711	1.0000	0.9441	0.9835	0.7793
ORR1D1|ERVL-MaLR	21490	1.0000	0.9441	0.9835	0.7793
ORR1D2|ERVL-MaLR	14702	1.0000	0.9441	0.9835	0.7793
ORR1E|ERVL-MaLR	21752	1.0000	0.9441	0.9835	0.0513
ORR1F|ERVL-MaLR	22291	1.0000	0.9441	0.9835	0.3022
RLTR17B_Mm|ERVK	28460	1.0000	0.9441	0.9835	0.7793
RLTR20A4|ERVK	21113	1.0000	0.9441	0.9835	0.7793
RMER15|ERVL	16421	1.0000	0.9441	0.9835	0.7793
RMER17C|ERVK	7233	1.0000	0.9441	0.0000	0.0000

TSSs	number of repeats	eb	ssc	sc	rs
MT2B|ERVL	12124	1.0000	0.9397	0.9900	0.9991
MTC|ERVL-MaLR	26995	1.0000	0.9397	0.9900	0.9991
MTD|ERVL-MaLR	50330	1.0000	0.9397	0.9900	0.9991
MTE2a|ERVL-MaLR	15338	1.0000	0.9397	0.9900	0.9991
MTE2b|ERVL-MaLR	13628	1.0000	0.9397	0.0041	0.0206
MTEa|ERVL-MaLR	19293	1.0000	0.9397	0.9900	0.6224
ORR1A3-int|ERVL-MaLR	9537	1.0000	0.9397	0.9900	0.9991
ORR1B1-int|ERVL-MaLR	27711	1.0000	0.9397	0.9900	0.9991
ORR1D1|ERVL-MaLR	21490	1.0000	0.9397	0.9900	0.9991
ORR1D2|ERVL-MaLR	14702	1.0000	0.9397	0.9900	0.9991
ORR1E|ERVL-MaLR	21752	1.0000	0.9397	0.0020	0.0102
ORR1F|ERVL-MaLR	22291	1.0000	0.9397	0.4781	0.0024
RLTR17B_Mm|ERVK	28460	1.0000	0.9397	0.9900	0.9991
RLTR20A4|ERVK	21113	1.0000	0.9397	0.9900	0.9991
RMER15|ERVL	16421	1.0000	0.9397	0.9900	0.9991
RMER17C|ERVK	7233	1.0000	0.9397	0.0000	0.0000

LINE and LTR family enrichments

Mouse: 0.25 FPKM min cutoff: Non-coding promoter regions: Mouse - rat matched samples

Promoters	number of repeats	sc	rs	
CR1_group	10428	0.7914	0.1165	
ERV1_group	65012	1.0000	1.0000	
ERVK_group	303654	1.0000	1.0000	
ERVK?_group	5273	0.9121	0.0444	
ERVL_group	103894	0.6418	0.0391	
ERVL-MaLR_group	440643	1.0000	0.0391	
L1_group	763835	1.0000	1.0000	

L2_group	55141	0.0320	0.0391	

TSSs	number of repeats	sc	rs	
CR1_group	10428	1.0000	0.7715	
ERV1_group	65012	1.0000	1.0000	
ERVK_group	303654	1.0000	1.0000	
ERVK?_group	5273	0.0013	0.0002	
ERVL_group	103894	1.0000	0.4197	
ERVL-MaLR_group	440643	0.5285	0.0000	
L1_group	763835	1.0000	1.0000	
L2_group	55141	1.0000	0.7715	

Mouse: 1 FPKM min cutoff: Non-coding promoter regions – rat matched samples

Promoters	number of repeats	sc	rs	
CR1_group	10428	1.0000	0.0626	
ERV1_group	65012	1.0000	1.0000	
ERVK_group	303654	1.0000	1.0000	
ERVK?_group	5273	1.0000	0.0947	
ERVL_group	103894	1.0000	0.1518	
ERVL-MaLR_group	440643	1.0000	0.0011	
L1_group	763835	1.0000	1.0000	
L2_group	55141	0.2606	0.1164	

TSSs	number of repeats	sc	rs	
CR1_group	10428	1.0000	0.6588	
ERV1_group	65012	1.0000	1.0000	
ERVK_group	303654	1.0000	1.0000	
ERVK?_group	5273	0.0249	0.0000	
ERVL_group	103894	1.0000	0.6588	
ERVL-MaLR_group	440643	1.0000	0.0001	
L1_group	763835	1.0000	1.0000	
L2_group	55141	1.0000	0.6588	

Rat: 0.25 FPKM min cutoff: Non-coding promoter regions

Promoters	number of repeats	sc		
CR1_group	10514	0.7630		
ERV1_group	60845	0.1740		
ERVK_group	272591	1.0000		
ERVL_group	100367	0.7690		
ERVL-MaLR_group	419388	0.5701		
L1_group	797150	1.0000		
L2_group	53301	0.4103		

RTE-BovB_group	2008	1.0000		

TSSs	number of repeats	sc		
CR1_group	10514	1.0000		
ERV1_group	60845	1.0000		
ERVK_group	272591	1.0000		
ERVL_group	100367	1.0000		
ERVL-MaLR_group	419388	1.0000		
L1_group	797150	1.0000		
L2_group	53301	0.4803		
RTE-BovB_group	2008	0.4803		

Rat: 1 FPKM min cutoff: Non-coding promoter regions

Promoters	number of repeats	sc		
CR1_group	10514	0.9115		
ERV1_group	60845	0.0382		
ERVK_group	272591	1.0000		
ERVL_group	100367	1.0000		
ERVL-MaLR_group	419388	1.0000		
L1_group	797150	1.0000		
L2_group	53301	0.0410		
RTE-BovB_group	2008	1.0000		

TSSs	number of repeats	sc		
CR1_group	10514	1.0000		
ERV1_group	60845	0.8501		
ERVK_group	272591	1.0000		
ERVL_group	100367	1.0000		
ERVL-MaLR_group	419388	1.0000		
L1_group	797150	1.0000		
L2_group	53301	0.2467		
RTE-BovB_group	2008	1.0000		

Zebrafish: 0.25 FPKM min cutoff: Non-coding promoter regions

Promoters	number of repeats	testis		
DIRS_group	17193	0.9999		
ERV1_group	13745	0.9999		
Gypsy_group	30134	0.9999		
Gypsy?_group	8633	0.9999		
L1_group	8886	0.9999		
L2_group	69876	0.9999		
LTR_group	30740	0.9999		
Ngaro_group	28873	0.9999		
Rex-Babar_group	12640	0.9999		

TSSs	number of repeats	testis		
DIRS_group	17193	1.0000		
ERV1_group	13745	1.0000		
Gypsy_group	30134	1.0000		
Gypsy?_group	8633	1.0000		
L1_group	8886	1.0000		
L2_group	69876	1.0000		
LTR_group	30740	1.0000		
Ngaro_group	28873	1.0000		
Rex-Babar_group	12640	1.0000		

Zebrafish: 1 FPKM min cutoff: Non-coding promoter regions

Promoters	number of repeats	testis		
DIRS_group	17193	0.9993		
ERV1_group	13745	0.9993		
Gypsy_group	30134	0.9993		
Gypsy?_group	8633	0.9993		
L1_group	8886	0.9993		
L2_group	69876	0.9993		
LTR_group	30740	0.9993		
Ngaro_group	28873	0.9993		
Rex-Babar_group	12640	0.9993		

TSSs	number of repeats	testis		
DIRS_group	17193	1.0000		
ERV1_group	13745	1.0000		
Gypsy_group	30134	1.0000		
Gypsy?_group	8633	1.0000		
L1_group	8886	1.0000		
L2_group	69876	1.0000		
LTR_group	30740	1.0000		
Ngaro_group	28873	1.0000		
Rex-Babar_group	12640	1.0000		

Tables for the p-values for the enrichment of repeat elements in the promoters of lncRNA/non-coding loci. Tables are provided for repeat classes and a specified list of ERV sub-families in mouse samples and for repeat families across samples for the three species analysed as part of this study. Two sample specific expression thresholds are applied in each instance.

Table S3

GO term	Description	P-value	FDR q-value	Enrichment (N, B, n, b)
GO:0006955	immune response	5.55E-08	6.22E-04	2.72 (7118,172,518,34)
GO:0045087	innate immune response	8.86E-06	4.96E-02	2.86 (7118,101,518,21)
GO:0006952	defense response	1.03E-05	3.84E-02	2.16 (7118,223,518,35)
GO:0098542	defense response to other organism	3.60E-04	1.00E+00	2.68 (7118,77,518,15)
GO:0002819	regulation of adaptive immune response	5.18E-04	1.00E+00	3.15 (7118,48,518,11)
GO:0002823	negative regulation of adaptive immune response based on somatic recombination of immune receptors built from immunoglobulin superfamily domains	6.40E-04	1.00E+00	6.25 (7118,11,518,5)
GO:0009617	response to bacterium	6.70E-04	1.00E+00	3.27 (7118,42,518,10)
GO:0006959	humoral immune response	6.75E-04	9.45E-01	4.37 (7118,22,518,7)
GO:0002822	regulation of adaptive immune response based on somatic recombination of immune receptors built from immunoglobulin superfamily domains	8.18E-04	1.00E+00	3.20 (7118,43,518,10)

GO terms enriched amongst Ensembl genes associated with coding assembly transcripts with poorly conserved ORFs.

Table S4

GO term	Description	P-value	FDR q-value	Enrichment (N, B, n, b)
GO:0006955	immune response	8.78E-07	9.83E-03	3.14 (7118,172,303,23)
GO:0002823	negative regulation of adaptive immune response based on somatic recombination of immune receptors built from immunoglobulin superfamily domains	5.06E-05	2.83E-01	10.68 (7118,11,303,5)
GO:0006952	defense response	6.85E-05	2.56E-01	2.42 (7118,223,303,23)
GO:0045087	innate immune response	8.56E-05	2.40E-01	3.26 (7118,101,303,14)
GO:0002820	negative regulation of adaptive immune response	1.31E-04	2.94E-01	9.04 (7118,13,303,5)
GO:0002819	regulation of adaptive immune response	1.58E-04	2.94E-01	4.40 (7118,48,303,9)
GO:0002710	negative regulation of T cell mediated immunity	2.96E-04	4.73E-01	17.62 (7118,4,303,3)
GO:0002822	regulation of adaptive immune response based on somatic recombination of immune receptors built from immunoglobulin superfamily domains	3.87E-04	5.42E-01	4.37 (7118,43,303,8)
GO:0035036	sperm-egg recognition	5.48E-04	6.82E-01	6.91 (7118,17,303,5)
GO:2000344	positive regulation of acrosome reaction	7.17E-04	8.02E-01	14.10 (7118,5,303,3)
GO:0002252	immune effector process	7.48E-04	7.62E-01	2.78 (7118,110,303,13)
GO:0002707	negative regulation of lymphocyte mediated immunity	8.38E-04	7.82E-01	8.54 (7118,11,303,4)

GO terms enriched amongst Ensembl genes associated with coding assembly transcripts with poorly conserved ORFs with a larger proportion of bases evolving faster than expected, when compared to their own UTRs.

Table S5

GO term	Description	P-value	FDR q-value	Enrichment (N, B, n, b)
GO:2000649	regulation of sodium ion transmembrane transporter activity	9.43E-04	1.00E+00	8.64 (7118,16,206,4)

GO terms enriched amongst Ensembl genes associated with coding assembly transcripts with poorly conserved ORFs with a smaller proportion of bases evolving faster than expected, when compared to their own UTRs.

Table S6

lncRNA identifier	Class	No. TSSs	Cell type with highest expression	Max phyloCSF score lncRNA	Max CPAT score lncRNA	Transcript with longest ORF	Peptides identified	Associated ERV
clfu_l20_10_c154	intergenic	2	RStid	13.515	0.13104	TCONS_00093362	DVARCSVSR (250)	RLTR16C_MM---LTR/ERVK
clfu_l20_10_c784	intergenic	1	RStid	-12.922	0.04547	TCONS_00107503	MGGLMRALR (326)	RMER17C---LTR/ERVK
clfu_l20_10_c839	intergenic	1	RStid	1.863	0.14752	TCONS_00117434	QSPSMLR (137)	RMER17C---LTR/ERVK
clfu_l20_11_c1049	intergenic	1	RStid	-45.906	0.06632	TCONS_00140757	MLKSQDR (178)	RMER4B---LTR/ERVK
clfu_l20_12_c548	intron_sense	1	RStid	-5.102	0.0609	TCONS_00193683	AIPPPPTDQPSK (854)	MTEb---LTR/ERVL-MaLR
clfu_l20_13_c385	TSS_sense	1	RStid	4.276	0.07535	TCONS_00245378	TQKCQDK (618)	RMER17B2---LTR/ERVK
clfu_l20_14_c229	TSS_anti	2	RStid	-28.582	0.06147	TCONS_00283245	MNPEGGRTAIQK (652)	MTD---LTR/ERVL-MaLR
clfu_l20_16_c406	intron_both	1	RStid	-81.755	0.02157	TCONS_00351781	MPGKVLRSQR (679)	MT2C_Mm---LTR/ERVL
clfu_l20_16_c480	intergenic	1	RStid	4.288	0.18034	TCONS_00368377	RWGDLER (1357)	RMER17C---LTR/ERVK
clfu_l20_16_c491	TSS_sense	1	RStid	-131.188	0.05613	TCONS_00369249	QDLISWLR (589)	MLT1A---LTR/ERVL-MaLR
clfu_l20_17_c760	intergenic	1	RStid	11.54	0.40072	TCONS_00425379	QIRASACAVALFTVPSFR (212)	RLTR27---LTR/ERVK
clfu_l20_18_c371	intergenic	1	Scyte	-13.475	0.03785	TCONS_00439915	MAEVASFVNRVLSK (245)	MT2A---LTR/ERVL
clfu_l20_2_c794	intergenic	1	Scyte	-211.071	0.02211	TCONS_00511323	GDMRAIQLLK (866) and DKCILLK (896)	MLT1C---LTR/ERVL-MaLR
clfu_l20_2_c940	intergenic	1	RStid	9.412	0.08863	TCONS_00557197	EWLESQLR (406)	MTEb---LTR/ERVL-MaLR
clfu_l20_3_c672	TSS_sense	1	RStid	-159.975	0.13546	TCONS_00583823	QNLEGDMGLAGSRK (342)	MLT1E2---LTR/ERVL-MaLR
clfu_l20_5_c912	TSS_anti	1	RStid	-19.77	0.04291	TCONS_00687300	KDTGRMAEQLLQK (250)	RLTR51A_Mm---LTR/ERVK?
clfu_l20_6_c648	intron_sense	1	RStid	13.201	0.27792	TCONS_00746689	TTEMTQPR (419)	MT2A---LTR/ERVL
clfu_l20_7_c1075	intergenic	1	RStid	45.741	0.06923	TCONS_00822197	GGDVLRTAGVLECLR (356)	MTE2a---LTR/ERVL-MaLR
clfu_l20_8_c767	TSS_both	1	Scyte	-22.925	0.05932	TCONS_00861992	NNFVKAHMR (364)	MTC---LTR/ERVL-MaLR
clfu_l20_9_c788	TES_anti	1	Scyte	-6.342	0.00514	TCONS_00913777	MCIRACK (617)	ORR1B1-int---LTR/ERVL-MaLR
clfu_l20_12_c335	intergenic	1	RStid	24.879	0.3576	TCONS_00177356	MPSSAGWTRMVMDEEQK (957)	RMER10B---LTR/ERVL
clfu_l20_15_c366	intergenic	1	RStid	-13.79	0.04598	TCONS_00321396	MRLHPAVEDLQEEGR (992)	RMER17A2---LTR/ERVK
clfu_l20_4_c699	intergenic	2	RStid	-0.048	0.08881	TCONS_00658670	DQWKMMK (300) and MMKQTIK (312)	IAPEY_LTR---LTR/ERVK

Annotation of lncRNAs for which open reading frames have been identified with peptides confirmed by mass spectroscopy. Numbers in brackets in the "Peptides identified" column represent the peptide start coordinate within the associated transcript.

Appendix Figure Legends and Figures

Appendix Figure S1

A) Overview of the computational pipeline used to detect and discover transcripts across replicates for the four samples used. **B)** Number of fragments filtered according to the Jaccard threshold used. The x-axis represents the Jaccard Score. The y-axis represents the proportion of the transcript set discarded. Data are shown for all transcripts (red) and those with high degrees of evidence (CAGE and polyA data, blue). The black line illustrates the difference between the two, used to select the threshold. **C)** Absolute number of transcripts retained in each set for sequential Jaccard Score sum thresholds. **D)** Complete (inset) and zoomed view of the cumulative frequency of annotated transcripts according to PhlyoCSF score. Colours represent the linked Ensembl annotation Biotype (see legend). **E)** Comparison between PhyloCSF score (x-axis) and $-\log_{10}$ of their BLASTx E-value against Ensembl and PfamA, and B. Each dot represents a transcript cluster coloured according to Ensembl biotype where available (see legend). The x-axis is limited to the region of interest.

Appendix Figure S2

Assembled transcript clusters that overlap pseudogene loci. **A)** The proportion of coding, non-coding and intermediate transcript clusters that overlap Ensembl annotated pseudogenes. **B)** The proportion of non-coding clusters with one or more promoter or TSS associated ERV that overlap pseudogene loci. **C)** Proportions are also given for a set of ERV subfamilies as representative examples. Clusters are assigned to the TSS associated set in preference.

Appendix Figure S3

A) The cumulative distance between each coding and non-coding TSS and the nearest CAGE peak as annotated by RIKEN 5 for both Ensembl genes and assembled clusters. **B)** The distance between the assembly TSSs from coding loci and the nearest Ensembl protein coding TSS. Assembly TSSs are divided according to their relative expression quantiles. Within each quantile the proportion of TSSs derived from non-coding loci that fall between the same thresholds are noted.

Appendix Figure S4

A) Similarly, to Figure 1, the genomic context of transcripts are displayed according to the cell type where their maximal expression was observed. **B)** Heatmaps for normalised variance stabilised transformed (VST) expression for both protein-coding transcripts and long non-coding RNAs across the three germline cell types. Expression levels are calculated from the median across replicates. **C)** The proportion of promoters containing an ERV from each subfamily that are assigned as non-coding (max PhyloCSF score <50)

compared to a set of all ERVs containing promoters and promoters with no ERVs. In this case a redundant (overlapping) promoter set was used. Promoter sets with more than 50 members are shown. **D)** Non-coding cluster classes divided according to whether a promoter associated with the cluster contains one or more ERV-element from each sub-family. Clusters can be represented in multiple pie charts

Appendix Figure S5

The expression of ERV associated lncRNAs. The relative expression level of lncRNA associated with promoter or TSS ERVs is demonstrated in each cell type; EB, SSC, Scytes and Rstids (panels **A-D** respectively, left). Also, shown (panels **A-D**, right) is the proportion of lncRNAs with one or more promoter associated ERV at two different expression thresholds (0.25 and 1.0).

Appendix Figure S6

The proportion of coding, non-coding and intermediate clusters with one or more promoter or TSS overlapping an ERV from each ERV subfamily of interest. TSS overlaps are depicted in preference to promoter overlaps.

Appendix Figure S7

The expression of CAGE tags from adult testes that uniquely map to promoter associated ERV elements for a set of ERV sub-families. ERV elements are tagged according to whether they are in the lncRNA associated, intermediate or background sets (Fig. 2C). Elements are divided according to whether they overlap a TSS and expression is divided according to the relative orientation of the CAGE tag with respect to the paired TSS. Coding and non-coding TSSs are considered separately. ERV classifications are shown. Numbers refer to the number of ERV-TSS pairs in each subset.

Appendix Figure S8

A) Coverage across individual promoter associated ORR1E LTRs, either overlapping the transcript TSS (Overlapping) or from elsewhere in the promoter (Non Overlapping). Each row represents an individual repeat in the first replicate of the 4 cell types studied. LTRs are scaled to the same length while the coverage of the 2kb flanking regions is also included. Coverage on the same strand (“Sense”) and the opposite strand (“Antisense”) relative to the corresponding TSS are depicted separately. **B)** Gradient coefficients of the mean coverage calculated across all promoter associated ERV sub-families of interest (Fig. 2C). ERVs are divided based on two criteria: i) whether they are overlapping a TSS (blue) or from elsewhere in the promoter region (non-overlapping, red); ii) the ERV classification based on non-coding promoter enrichment (Fig. 2C, rows). The y-axis represents the gradient of the reads orientated in the direction of the neighbouring transcripts (sense). The x-axis corresponds to the gradient of reads orientated in the

opposite direction (antisense). Each biological replicate from the 4 cell types is plotted individually (columns). **C)** Comparison of the number of non-coding to coding promoter regions associated with individual repeat element sub-families amongst transcript clusters expressed in mouse round spermatids. Spot colour corresponds to the class of each repeat. Spot size corresponds to the significance of the non-coding enrichment. The black line records the background proportion of expressed promoter regions. **D-F)** Coverage plots for the most highly enriched promoter associated repeat family from the DNA class in each of the three species, data is represented as shown previously (Figure 4A).

Appendix Figure S9

A) Scatterplot indicating the fraction of tissue-specific (τ >= 0.75) lncRNA transcripts from mouse ENCODE with a unique ERV element associated versus the total number of tissue specific lncRNA transcripts for ERVs that directly overlap annotated TSSs and **(B)** those within 1000bp upstream of the TSS. Clearly, testis is an outlier in both instances with either the highest or second highest proportion of ERV associated tissue-specific lncRNAs and in terms of raw number of ERV associated ERVs. Raw counts of unique tissue-specific ERV associated lncRNAs are shown. **C)** Raw counts for ERVs directly overlapping TSSs and **D)** those within 1000nt upstream. **E)** The expression in $\log_{10}$(FPKM) of lncRNAs and protein coding transcripts with ERVs in either their promoter (1000bp) or overlapping their TSS across the thirteen tissues.

Appendix Figure S10

A) Heatmap illustrating the counts of the most abundant unique ERV subfamilies associated with tissue-specific lncRNA transcripts (tissue specificity score τ >= 0.75) based on ERVs within 1kb of the annotated TSS and for ERVs **(B)** that overlap the annotated TSS. Expression is judged using 13 mouse encode tissue RNAseq datasets.

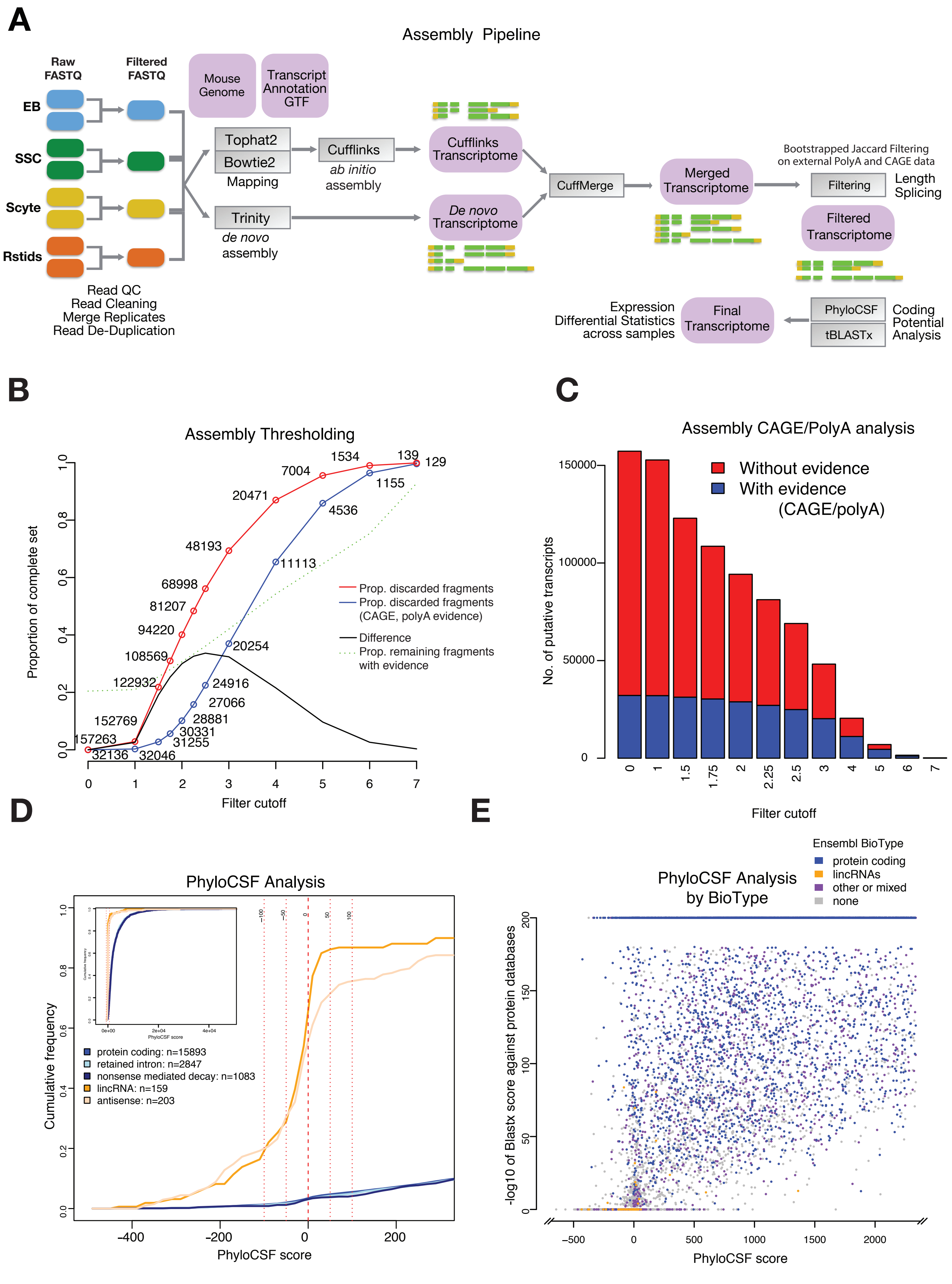

Figure S1: Davis *et al*, 2017

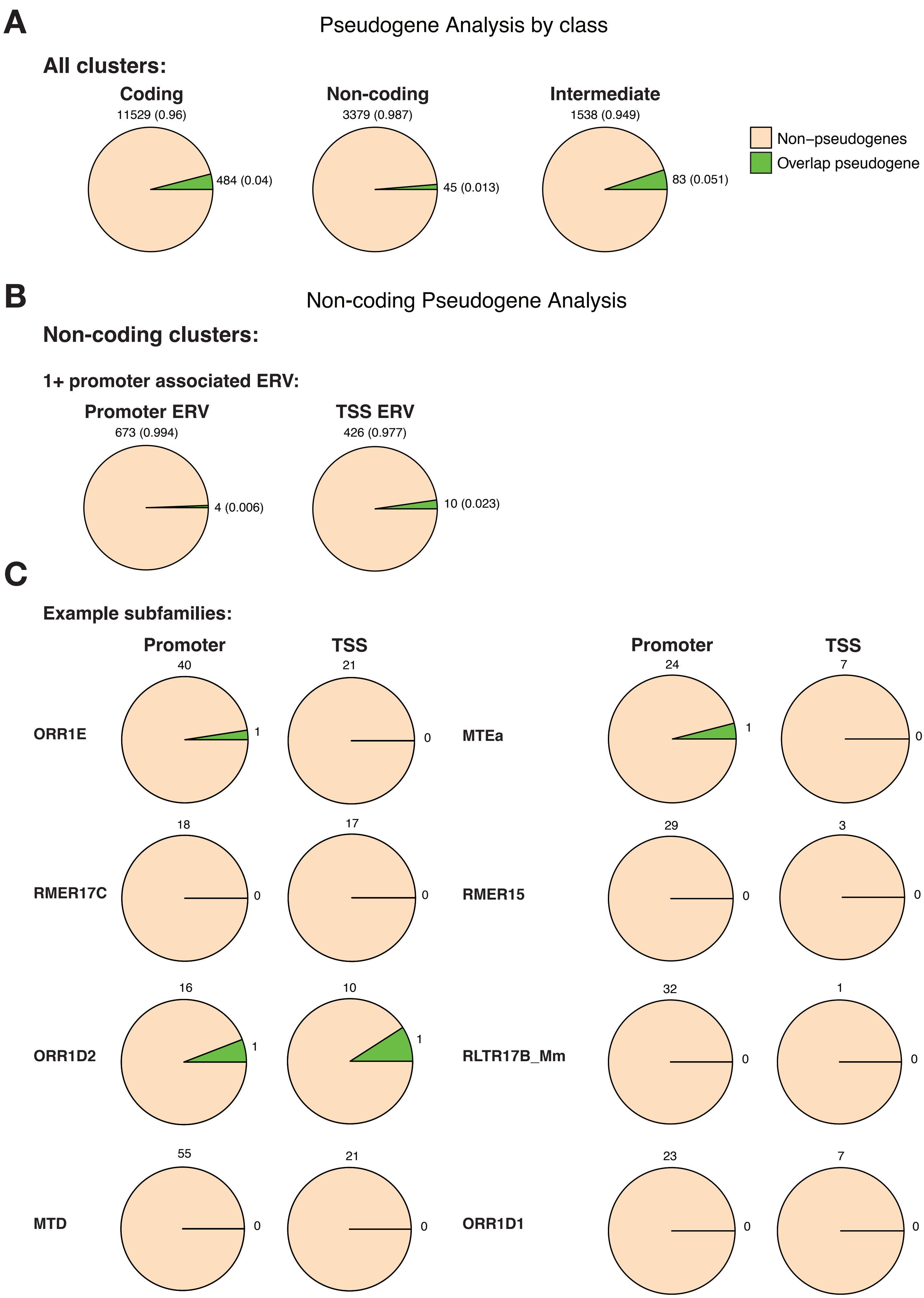

Figure S2: Davis *et al*, 2017

A

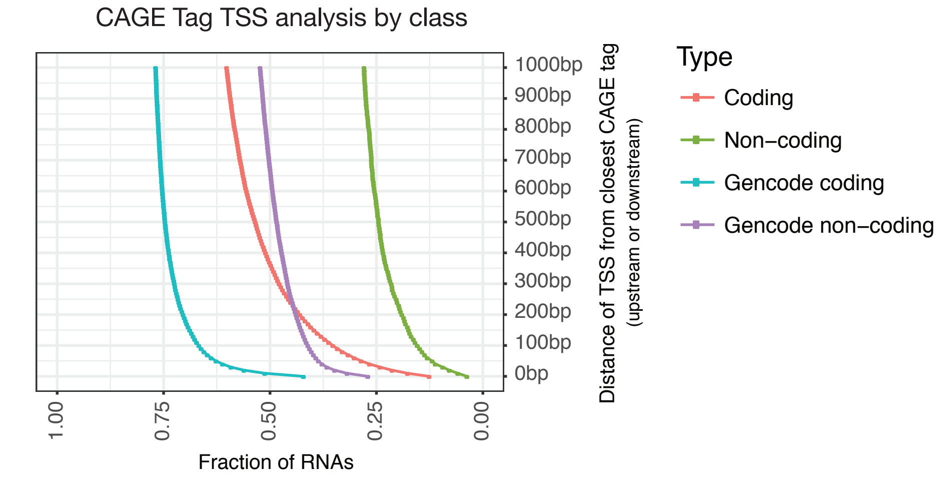

B

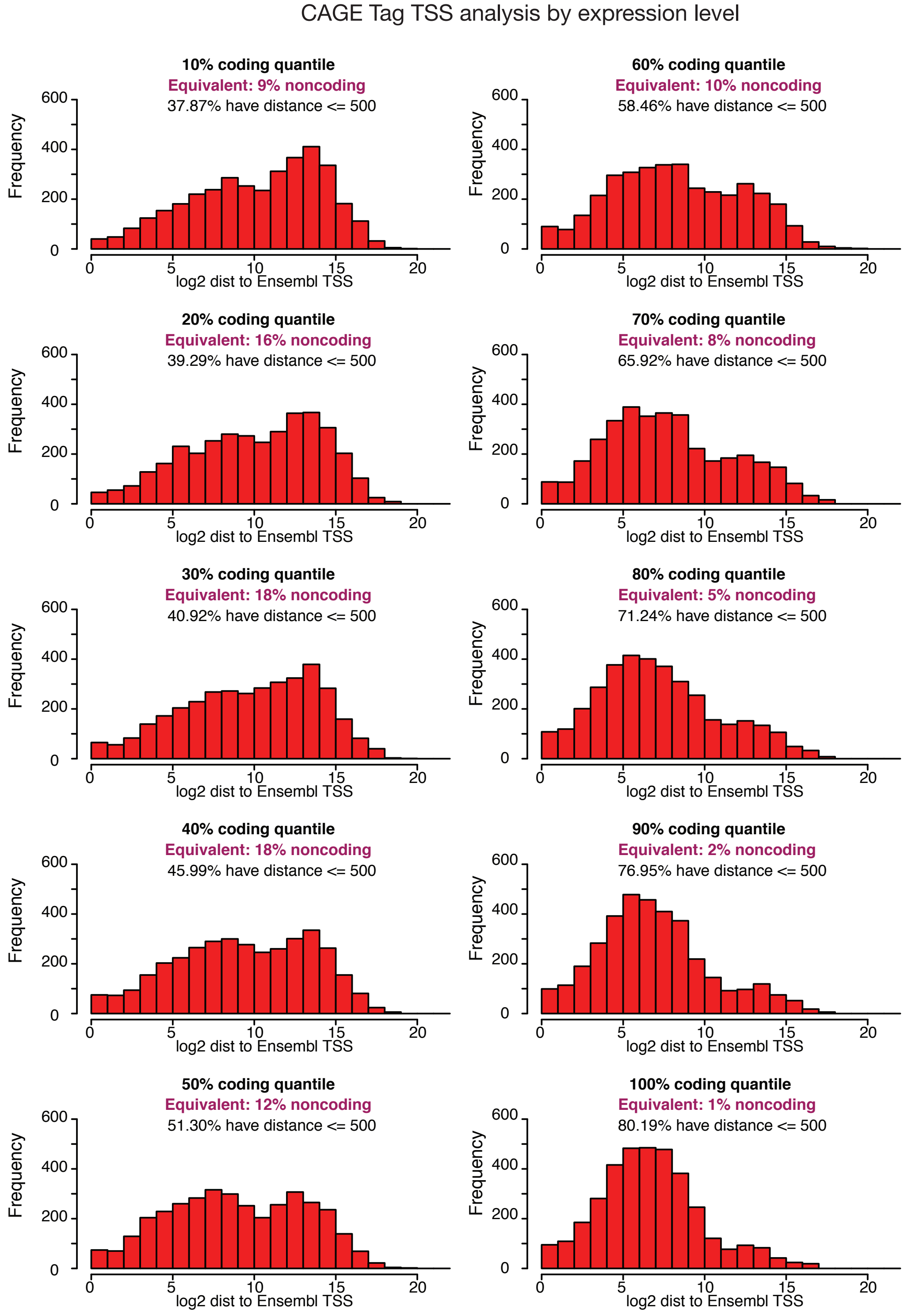

Figure S3: Davis *et al*, 2017

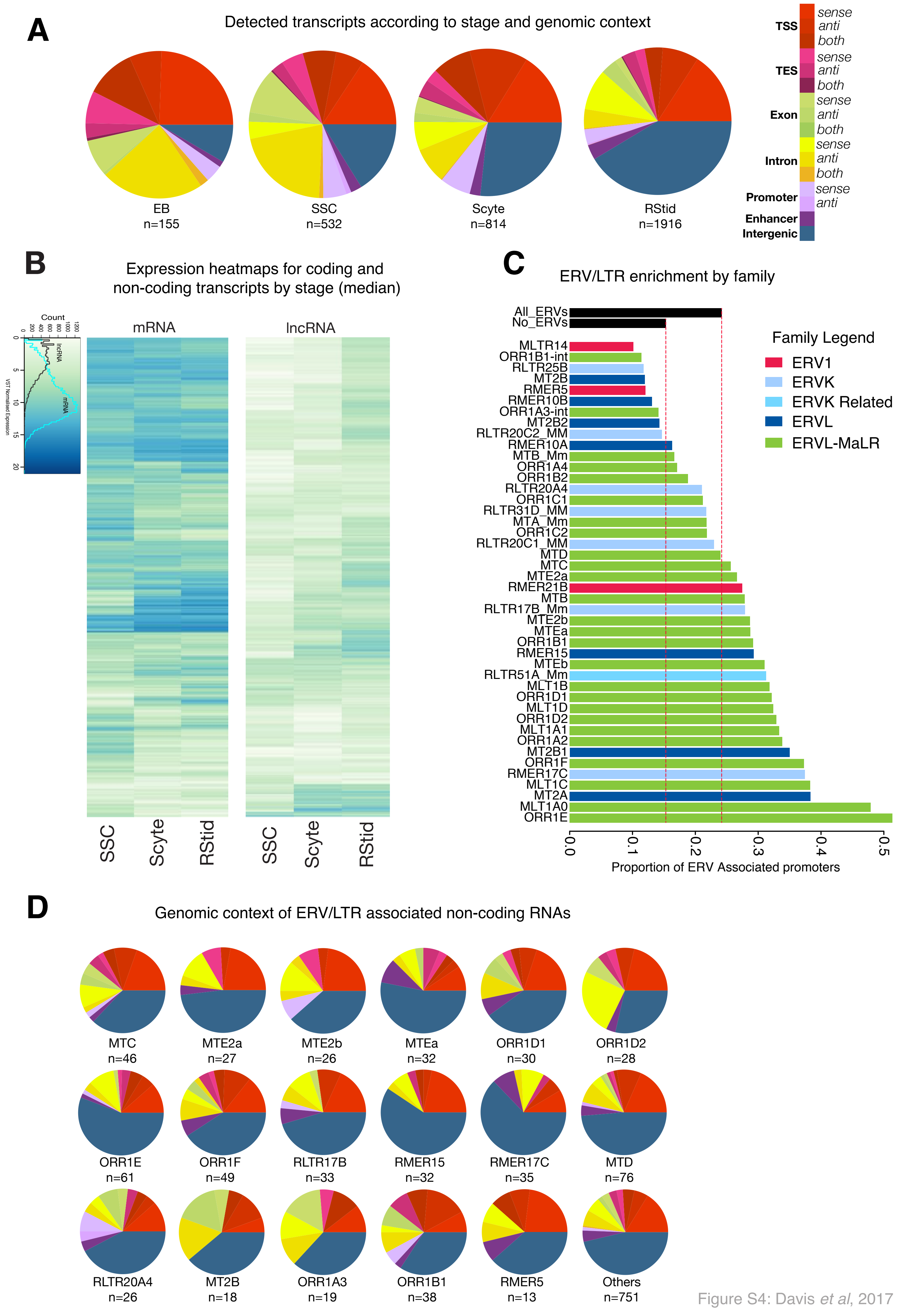

Figure S4: Davis *et al*, 2017

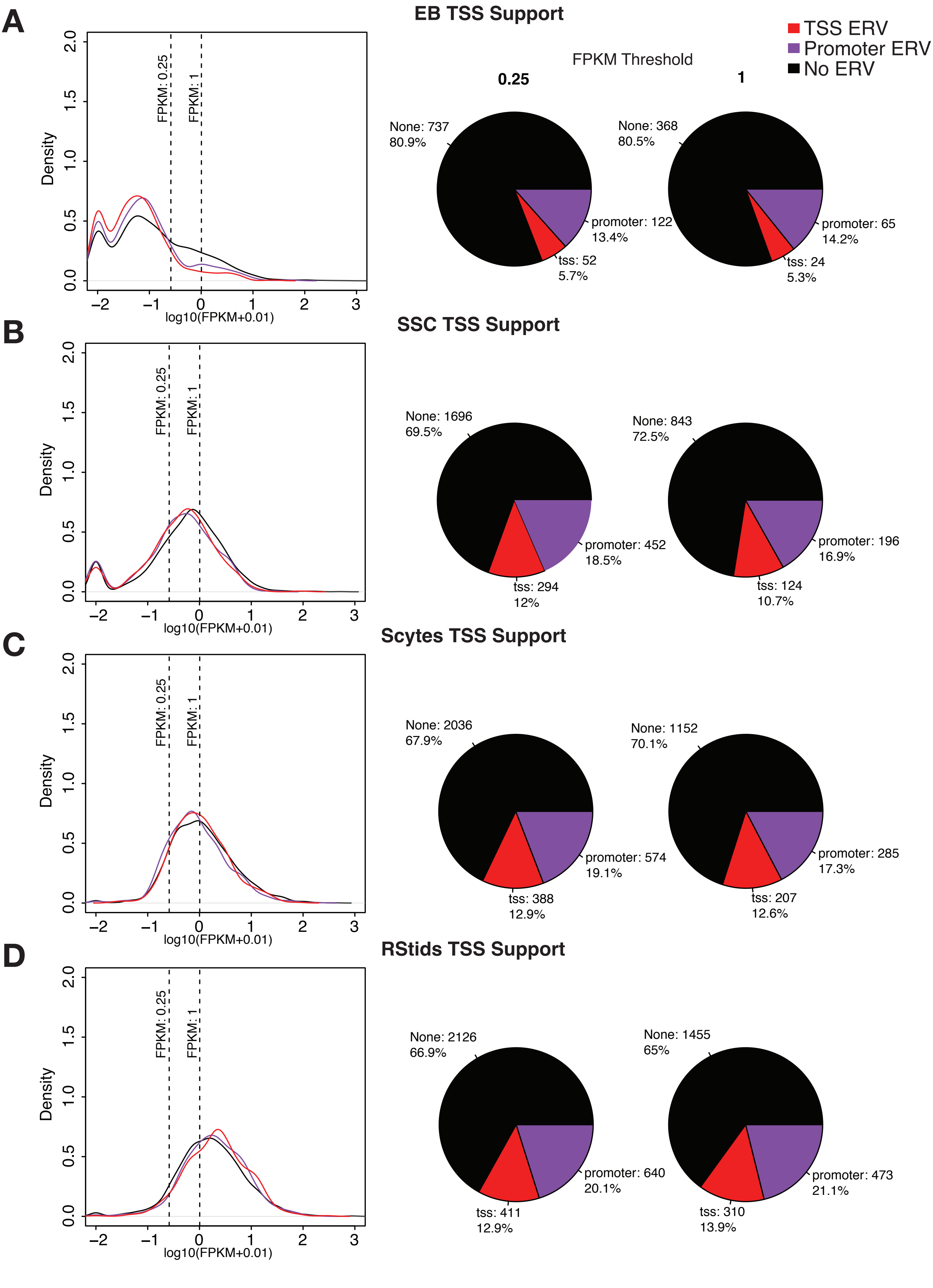

Figure S5: Davis *et al*, 2017

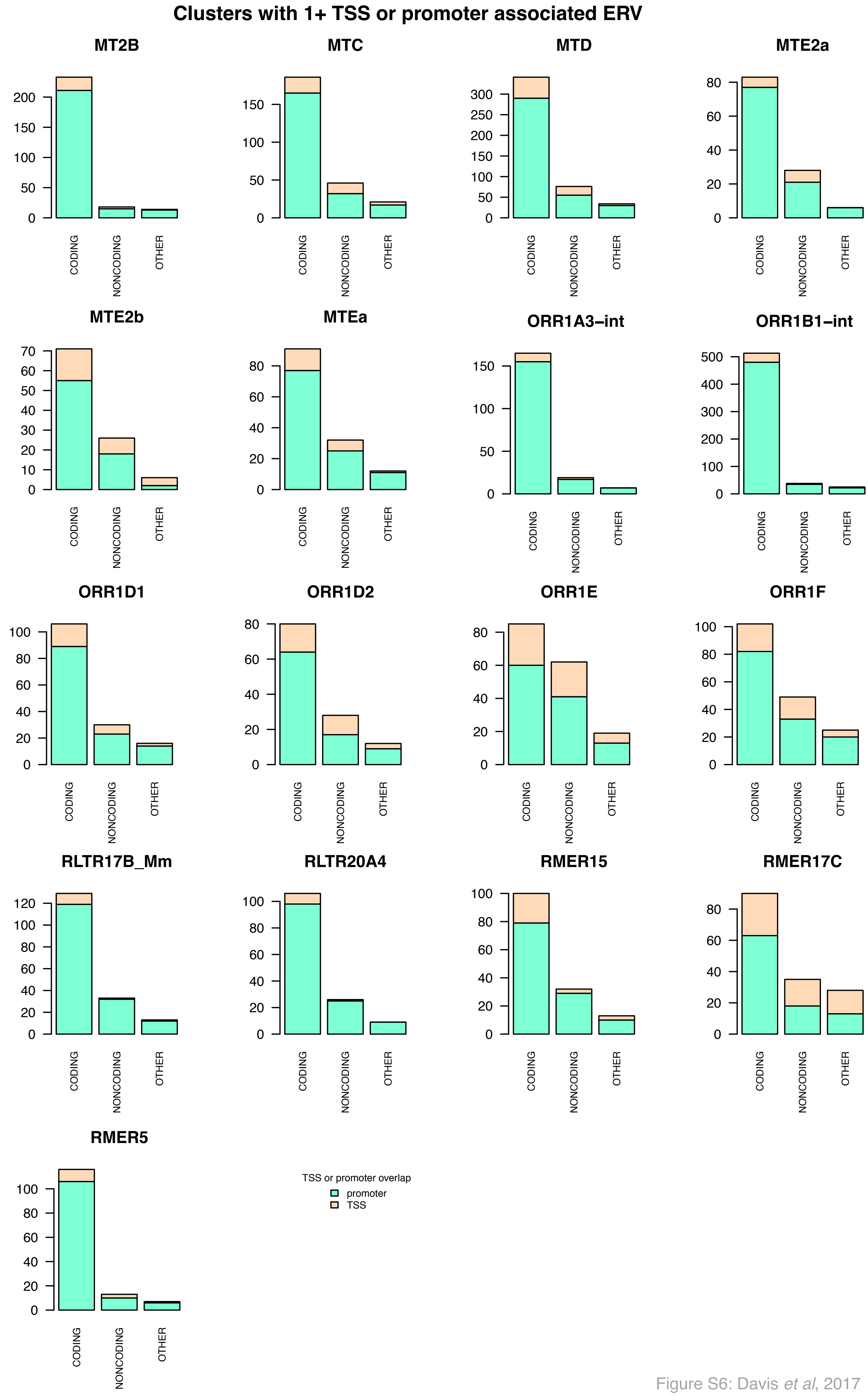

Figure S6: Davis *et al*, 2017

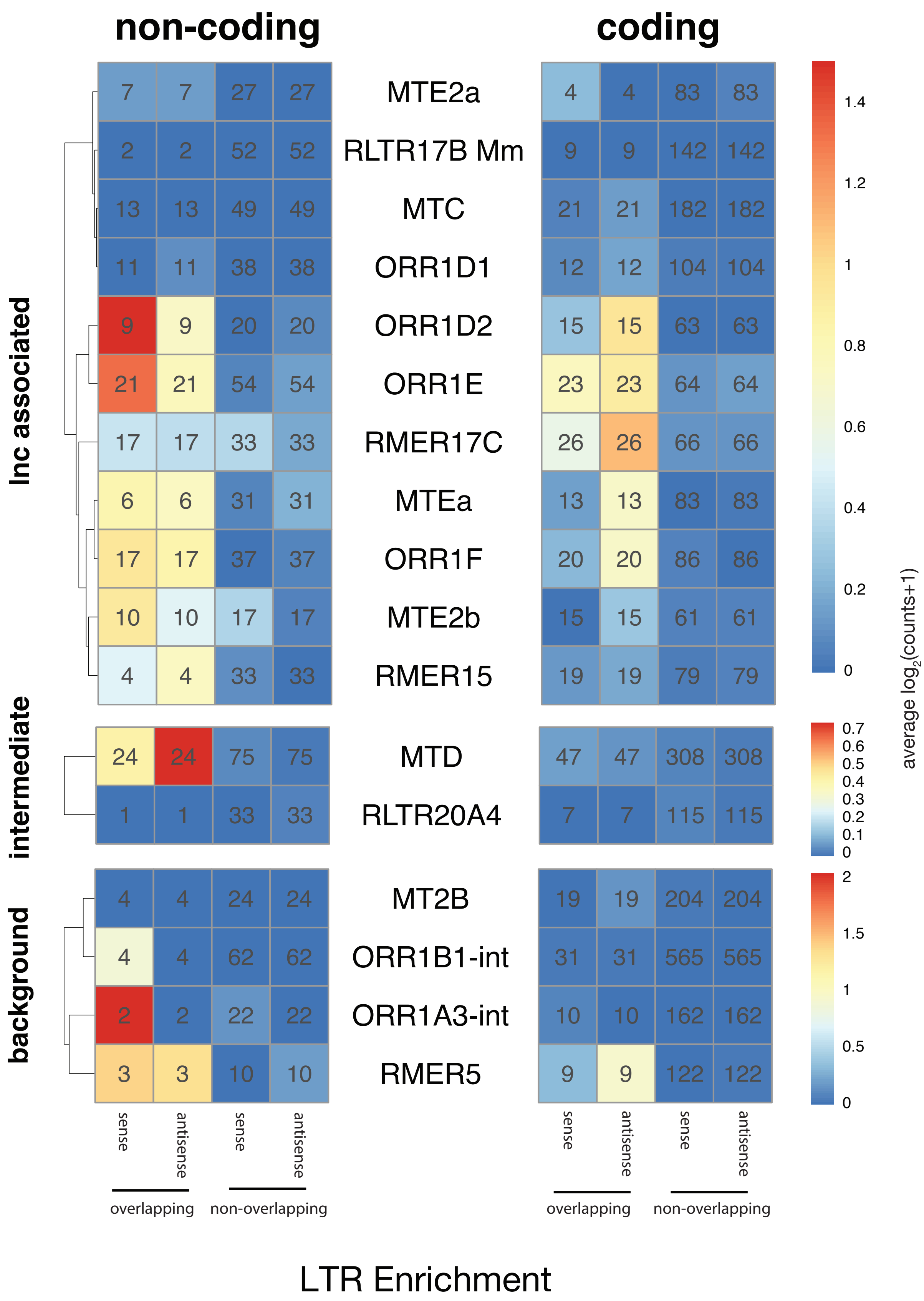
non-coding
coding
lnc associated
intermediate
background
MTE2a
RLTR17B Mm
MTC
ORR1D1
ORR1D2
ORR1E
RMER17C
MTEa
ORR1F
MTE2b
RMER15
MTD
RLTR20A4
MT2B
ORR1B1-int
ORR1A3-int
RMER5
7 7 27 27
2 2 52 52
13 13 49 49
11 11 38 38
9 9 20 20
21 21 54 54
17 17 33 33
6 6 31 31
17 17 37 37
10 10 17 17
4 4 33 33
24 24 75 75
1 1 33 33
4 4 24 24
4 4 62 62
2 2 22 22
3 3 10 10
4 4 83 83
9 9 142 142
21 21 182 182
12 12 104 104
15 15 63 63
23 23 64 64
26 26 66 66
13 13 83 83
20 20 86 86
15 15 61 61
19 19 79 79
47 47 308 308
7 7 115 115
19 19 204 204
31 31 565 565
10 10 162 162
9 9 122 122
sense
antisense
sense
antisense
overlapping
non-overlapping
average log2(counts+1)
1.4
1.2
1
0.8
0.6
0.4
0.2
0
0.7
0.6
0.5
0.4
0.3
0.2
0.1
0
2
1.5
1
0.5
0
LTR Enrichment

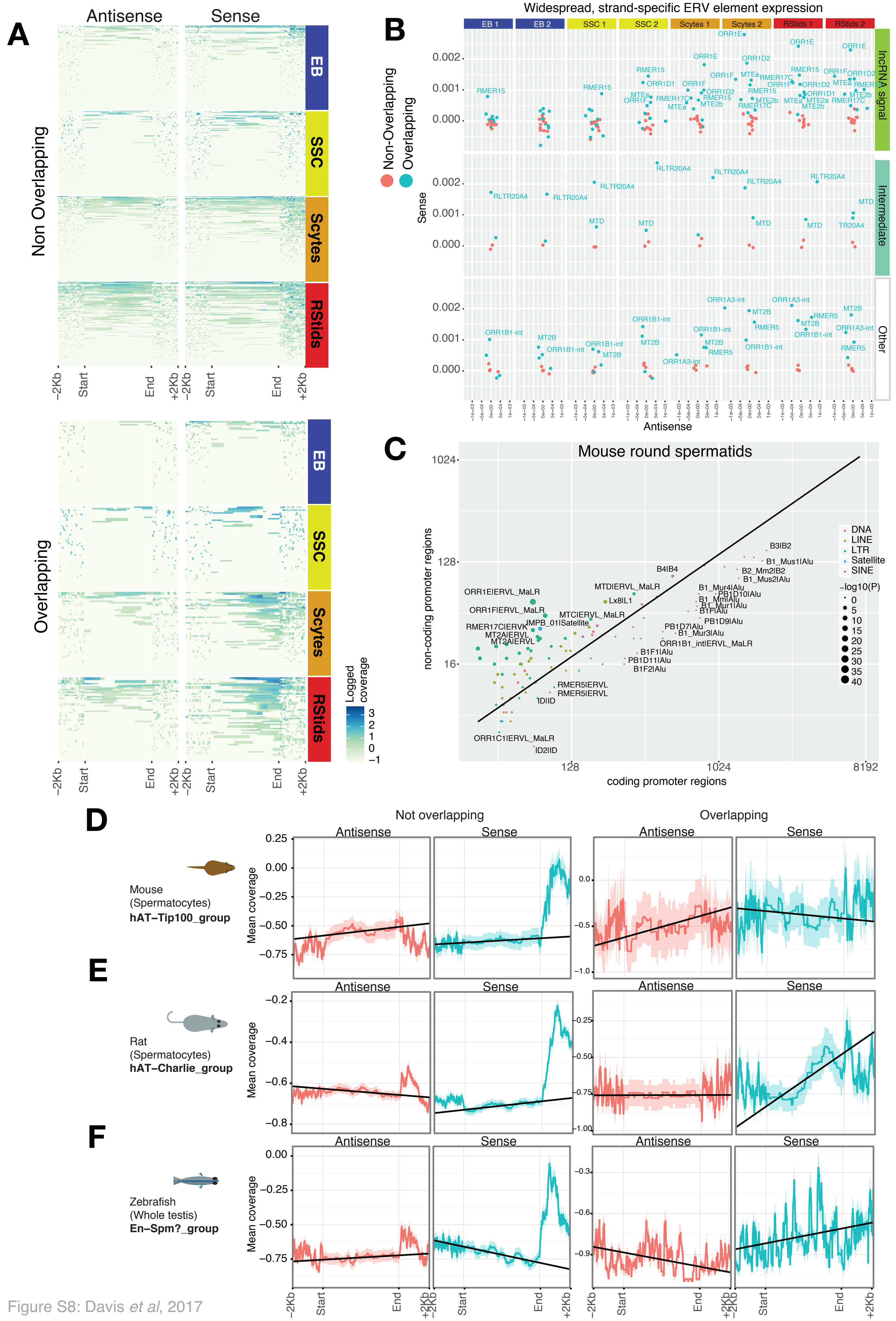

Figure S8: Davis *et al*, 2017

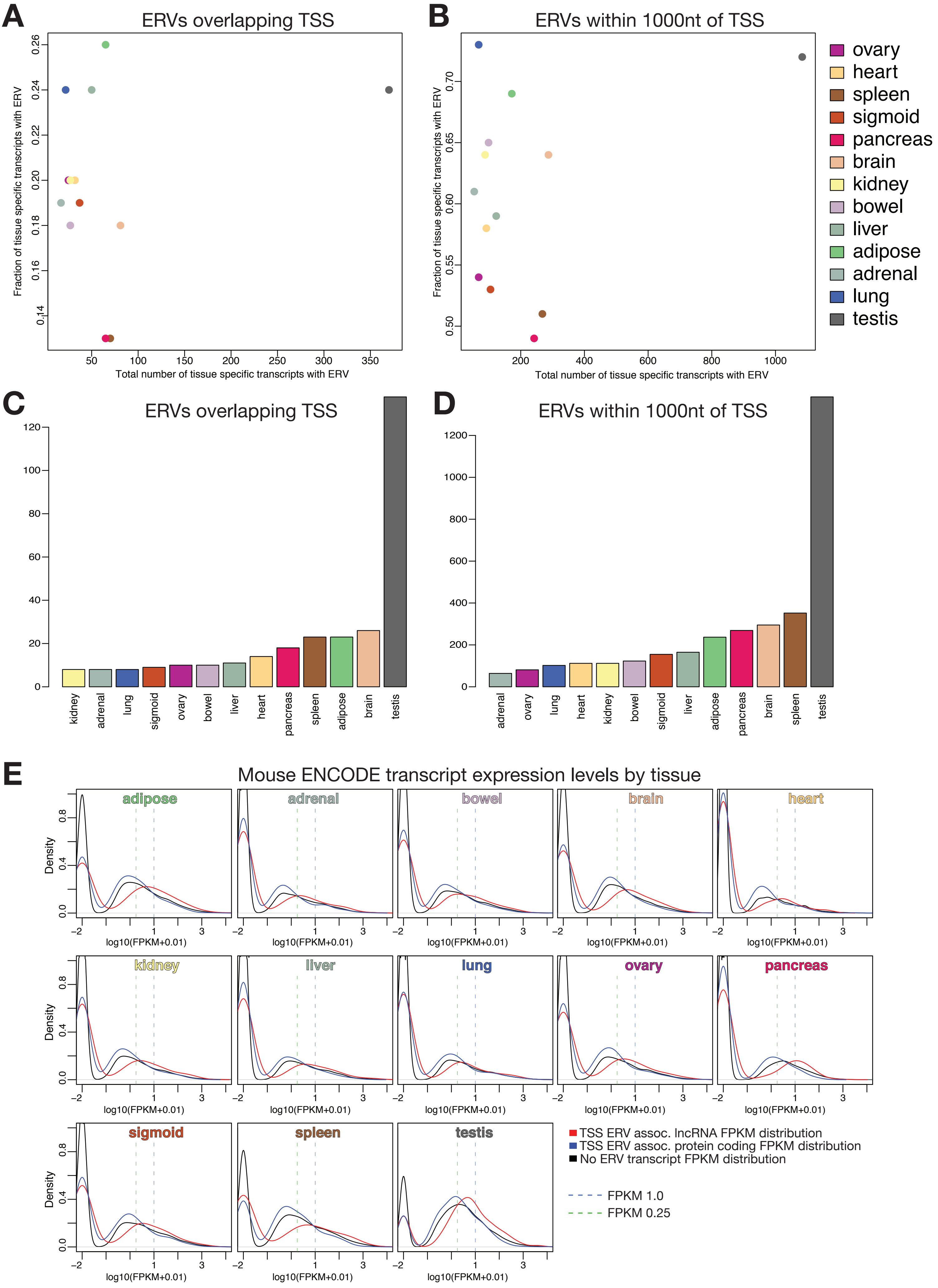

Figure S9: Davis *et al*, 2017

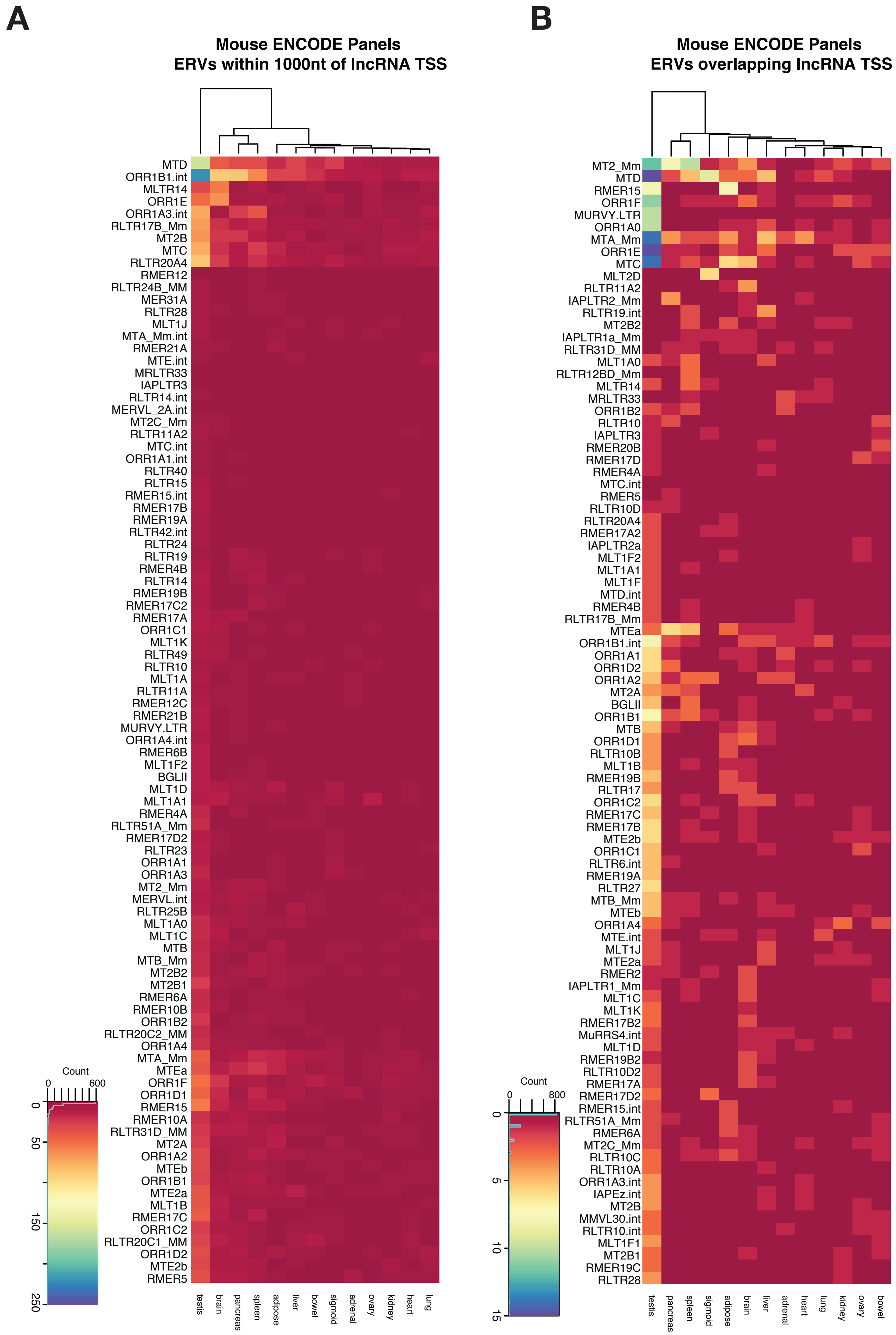

Figure S10: Davis *et al*, 2017

Appendix methods

Preparation of samples

For the primary transcriptional analysis, cells from each of the four developmental stages (EB, SSC, Scytes and Rstids), in mouse (C57BL/6N) were prepared as two independent biological replicates as follows. Erythroblasts were differentiated from E12.5 mouse livers *in-vitro* [1, 2]. SSCs were cultured *in-vitro* as described previously [3]. *Ex-vivo* germ cells were obtained from dissection of 8 weeks old adult mice through enzymatic digestion and mechanical disaggregation in EKRB buffer as previously described [4]. Mouse spermatocytes and round spermatids were isolated and purified through Becton-Dickinson Aria II cell sorter upon staining with Hoechst DNA dye as previously published [5].

For the cross-species analysis, adult rat spermatocytes were obtained and purified through a similar procedure to above and paired with additional mouse spermatocytes and round spermatids, identically prepared to provide matched data. Zebrafish were raised and maintained using standard procedures [6] with whole testes dissected from one-year-old adult male Zebrafish, with each replicate prepared from 8 testes. Mice used were inbreed C57BL/6N strain, rats were Sprague Dawley® strain purchased by Charles River. Zebrafish used were standard wildtype AB. Mice and rats were maintained at the EMBL Mouse Biology Unit, Monterotondo in accordance with current Italian legislation (Art. 9, 27 January 1992, number 116) under license from the Italian health ministry.

Preparation of RNA sequencing libraries

RNA from the above samples was prepared via Qiazol lysis followed by DNAse treatment in the presence of an RNAse inhibitor (RNaseOUT™, Invitrogen). RNA was recovered via EtOH precipitation. 5µg of this RNA were ribodepleted using Ribo-Zero-rRNA Removal Kits (Ribo-Zero™, Illumina) and 50ng of this RNA was used for strand-specific cDNA library preparation (ScriptSeq RNA-Seq Library Prep Kit, Illumina). Libraries were purified and analyzed on High Sensitivity DNA chip (Agilent) on BioAnalyzer and each library was sequenced one lane of a HiSeq 2000 sequencer.

Transcriptome assembly

The 8 primary samples above, were used to assemble the transcriptome (2 each from EBs, SSCs, Scytes and RStids). Fastq files were cleaned, adapters were trimmed and redundancy was removed using *Reaper* and *Tally* within the *SequenceImp* pipeline [7], maintaining FASTQ format (additional *Reaper* options: `-clean-length 50 -mr-tabu 14/2/1 -3p-global 14/1/0 -3p-prefix 10/2/1/0 -3p-head-to-tail 0 -qqq-check 40/5/0/20 -nnn-check 2/9 -dust-suffix 20;`

additional *Tally* options: `--with-quality`). Following this, biological replicate data was concatenated and redundancy removed once more with *Tally*. Finally reads were renamed and numbered.

Transcriptomes were assembled independently for each cell type using *Cufflinks* [8] and *Trinity* [9]. For Cufflinks insert sizes were estimated and paired reads were aligned to the mouse genome (mm10) using TopHat2 [8] before assembly *(TopHat2* options: `--library-type fr-secondstrand --mate-inner-dist -47 --mate-std-dev 59 -p 8; Cufflinks options: --library-type fr-secondstrand`). For *Trinity*, sample reads were first assembled into transcripts (*Trinity* options: `--seqType fq --SS_lib_type FR --no_run_butterfly`) and then aligned to the genome using *gmap* (*gmap* options: `-f 2 -n 1 --quiet-if-excessive -x 50 -t 6`). Finally the 8 assemblies were merged using *Cuffmerge*. The merged assembly was filtered to remove all transcripts with a length less that 200nt, those without one or more intron and those without strand information.

In order to enrich for transcripts for which the 5' and 3' ends are confidently identified, clustered polyA tags were retrieved from GEO, representing mouse brain, kidney, liver, muscle and testis (GSM747481, GSM747482, GSM747483, GSM747484, GSM747485) [10]. FANTOM CAGE tags were retrieved and prepared for FANTOM 3 and 4 using the *CAGEr* and *FANTOM3and4CAGE* Bioconductor packages. Mouse tissue data was imported using the groups: brain, cerebral_cortex, hippocampus, cerebellum, liver, lung, diencephalon, visual_cortex, testis, prostate_gland, heart, muscle, macrophage and samples: brain, cerebral_cortex, hippocampus, cerebellum, liver, control_mouse, diencephalon, visual_cortex, testis, prostate_gland, heart, muscle, unstimulated_sample. Tags were normalised using the power-law and clustered based on a distance threshold of 20. Singletons are filtered unless they possess a normalised value of 5 or more. Cumulative tag distribution calculations are used to calculate the promoter boundaries based on the 10th and 90th percentile of the CAGE signal. Consensus clusters are determined using a TPM threshold of 5 and a maximum distance of 100. Poly-A and CAGE annotation was lifted over from mm9 to mm10. The distance between each transcript TSS and the nearest CAGE tag was calculated along with the distance between the last transcript exon and the nearest downstream polyA site. polyA sites within the last exon were considered to have a distance of zero.

Cell specific CAGE and polyA data were not available. Therefore we designed an alternative approach to enrich the assembly for transcripts for which we are most confident. Each of the 8 cell line specific assemblies (4 Cufflinks and 4 Trinity) were compared to the merged assembly and for each transcript in the merged assembly the best Jaccard score for overlap with each sub-assembly was recorded (0-1) (see below).

A sum of the Jaccard scores across all 8 assemblies was determined for each merged transcript as a surrogate measure of cross-assembly evidence. A Jaccard Score sum threshold was chosen for transcript retention that maximized the difference between the proportion of the assembly discarded and the proportion of transcripts with end evidence discarded (CAGE within 1000bp of TSS and polyA signal within or 500bp downstream of the last exon) (Fig. S1B+C). A Jaccard sum threshold of 2.5 was applied as a filter.

Finally, transcripts residing on Ensembl supercontigs rather than chromosomes were discarded. In order to remove a level of redundancy in the downstream analyses, the filtered assembly transcripts were grouped according to their exonic overlap to generate a set of related, transcript clusters, that will, to an extent, share features and function. This was achieved by computing all pairwise Jaccard scores between all pairs of overlapping transcripts, resulting in a network representation of transcript overlap. This graph was clustered using mcl with default parameters, and the resulting transcript clusters were used.

Jaccard Score

At various points transcripts were classified and filtered based on their overlap with other transcripts. In order to do so, the Jaccard measure of overlap was used, ranging from zero to one. Between two transcripts, it is defined as the number of shared bases divided by the total number of unique bases within the two transcripts (thus bases in both transcripts are counted only once). For non-overlapping transcripts this measure yields 0, for identical transcripts it yields 1.

Coding potential

The coding potential of transcripts was determined using *BLAST* [11] and *PhyloCSF* [12]. *PhyloCSF* scores were generated for each transcript from a 29 species alignments in MAF format (Options: `--orf=StopStop3 --frames 3 –removeRefGaps --minCodons=25`). Alignment files were downloaded from UCSC [13]:
http://hgdownload.cse.ucsc.edu/goldenPath/mm10/multiz60way/maf/
and parsed to concatenate exonic regions. Species selected included mouse (mm10), rat (rn5), Guinea pig (cavPor3), rabbit (oryCun2), human (hg19), chimp (panTro4), rhesus (rheMac3), bush baby (otoGar3), tree shrew (tupBel1), shrew (sorAra1), hedgehog (eriEur1), dog (canFam3), cat (felCat5), horse (equCab2), cow (bosTau7), armadillo (dasNov3) elephant (loxAfr3), tenrec (echTel1), tarsier (tarSyr1), mouse lemur (micMur), kangaroo rat (dipOrd1), squirrel (speTri2), pika (ochPri2), alpaca (vicPac1), dolphin (turTru2), microbat (myoLuc2), megabat (pteVam1), rock hyrax (proCap1) and sloth (choHof1). BLASTx (ncbi-blast-2.2.28+) compared the assembled transcript sequences against Ensembl mouse v69 proteins and Pfam A and B [14] databases

(Pfam v27.0 90% non-redundant with segmasker data added) (Options: `-db_soft_mask 21 -outfmt 6 -max_target_seqs 1 -strand plus –evalue 0.01`). In each case, where a transcript is associated with multiple protein alignments, the alignment with the lowest E-value is selected. For each assembly cluster a maximum phyloCSF score and a maximum $-\log_{10}$(e-value) for BLASTx across all databases was calculated. Where one or more transcript from within a cluster was missing a phyloCSF score, a large out of bounds value was assigned. Transcripts with no blast hits against a database were assigned a $-\log_{10}$(e-value) of 0. E-values of 0 were assigned a $-\log_{10}$(E-value) score of 200. Maximum score thresholds of 10 for the cluster $-\log_{10}$(BLASTx E-value) and 50 for the cluster phyloCSF score were chosen to discriminate "coding" clusters from "non-coding" clusters. Intermediate classes of clusters failed one of these criteria. Where used, CPAT [15] scores were calculated with CPAT v1.2.1 and the maximum CPAT score was calculated per cluster.

Selection of coding potential thresholds

The phyloCSF threshold was selected to clearly separate those transcripts associated with Ensembl lincRNA annotation from those overlapping protein coding transcripts. Assembly transcripts are paired with Ensembl transcripts according to those that share the greatest exonic overlap, other transcripts are discarded. Each assembly transcript is assigned the corresponding Ensembl biotype (v69). Transcripts for which a phyloCSF score could not be assigned were also discarded (Fig. S1D). This threshold was confirmed at the cluster level and a maximum BLASTx threshold was chosen by assigning transcript clusters a corresponding Ensembl biotype. Clusters are assigned biotypes based on the jaccard score for a same strand overlap between the assembly transcripts and Ensembl annotation (v69) (cutoff = 0.333). Where no Blast hits were found an e-value of 1 was assigned. Infinite $-\log_{10}$(min. E-values) were assigned an *out of bounds* value of 200. Only clusters where a phyloCSF score could be assigned, are plotted. The scores were chosen as they clearly demarked the majority of clusters found to represent Ensembl lincRNAs from those representing protein-coding genes (Fig. S1E).

Cluster expression

Unless otherwise stated for individual analyses, transcript cluster FPKM expression profiles were determined by RSEM [16] (Options: *--paired-end --strand-specific*). The mean expression was taken between biological replicates. Clusters were defined as "expressed" in a cell type using a minimum mean expression threshold of 0.25 FPKM. Cell lines within which a cluster is most highly expressed were determined using the mean FPKM values. Clusters with tied expression were discarded from these analyses.

Exon conservation

The multiple alignments of 59 vertebrate genomes to the mouse genome mm10 (GRCm38) were used to compute the *PhyloP* [17] scores. The file "mm10.60way.phyloP60way.bw" was downloaded from http://hgdownload.cse.ucsc.edu/goldenPath/mm10/phyloP60way/. The plot was generated utilizing *deepTools* [18] version 1.5.9.1. For each cluster only the exons of the longest transcript are included. Each column within the heatmap represents a bin of 10 bp. All exons are scaled to a length of 1,000 bp.

LncRNA conservation and exonic repeat composition

For each non-coding cluster the longest isoform was screened against 15 genomes (chimpanzee, cow, gorilla, horse, human, macaque, megabat, microbat, mouse, opossum, pig, rabbit, rat, squirrel and Tasmanian devil) corresponding to Ensembl (v69). lncRNAs were mapped as described in previous reports [19, 20]. In brief, each mouse query was compared to the genomes WU-Blast [21] version 2.0MP (04-May-2006) (Options: `-e 0.00001 -filter=seg –lcfilter`). The identified genomic regions were sorted by increasing BLAST E-values and re-aligned with *Exonerate* [22] version 2.2.0. The first region resulting in a successful *Exonerate* alignment (spanning at least 70% of the original query) was kept. These putative homologues for each query were considered for multiple sequence alignment with *T_coffee* [23] version_9.03.r1318 (2012-07-12 19:05:45 - Revision 1318 - Build 366). The pairwise sequence identity between each putative homologue was then measured. For exonic repeat content, repeats were predicted with *repeatMasker* v4_0_6 (Options: `-a -xsmall -gff -html -e wublast -no_is`). The repeat and low complexity content of the mapped transcripts and those that did not map successfully to the genomes above was determined using the *RepeatMasker* [24] repeat and low-complexity content (Ensembl v69).

RNA polymerase II occupancy at transcript TSSs

Two replicates of RNA polymerase II ChIP-seq data and the associated input data were downloaded from ENCODE [25] for mouse (adult 8 weeks) testis:
http://hgdownload.cse.ucsc.edu/goldenPath/mm9/encodeDCC/wgEncodeLicrTfbs/

Reads from the two replicates were merged and mapped to the mouse genome (mm10) using *Bowtie 2* [26] (version 2.2.3, Option: `--very-sensitive`). *BedTools2* [27] *genomecov* (version v2.22.1) and the UCSC tool *bedGraphToBigWig* were used to determine digital counts for each nucleotide in the genome. Counts were scaled according to library size and the input was subtracted using *deepTools bigwigCompare* (version 1.5.9.1) [18]. The plot was generated with *deepTools*. Where multiple transcripts have a TSS within a 50 bp window, only the TSS of the longest isoform is retained.

Assessment of assembly 5' ends with FANTOM5 CAGE data

The mouse CAGE data in mm9 coordinates were downloaded from the RIKEN 5 portal and lifted over to mm10 coordinates using the liftOver tool with the chain files generated by the UCSC genome browser. We then used BedTools to find the closest peak to the TSS of each transcript from the assembled coding or non-coding clusters. As a control, we also identified the CAGE peaks closest to coding and non-coding TSSs annotated in Ensembl (v87). Coding genes were selected from Ensembl based on the presence of an annotated ORF, whereas non-coding genes were selected if they belonged to one of the following classes: *3prime_overlapping_ncRNA ambiguous_orf antisense antisense_RNA lincRNA ncrna_host non_coding non_stop_decay processed_transcript retained_intron sense_intronic* and *sense_overlapping*.

Assessment of assembly 5' ends with Ensembl annotation

The assembly transcript clusters were separated according to coding potential. Only those coding transcripts and clusters in our assembly which overlapped an Ensembl *protein_coding* locus in a strand specific manner were considered. Transcriptwise FPKM expression was determined using RSEM (v1.2.7) (Options: --paired-end --strand-specific) [16]. Transcripts were merged and their expression summed if they shared the same TSS. Transcripts from coding loci were separated into ten expression quantiles. Within each quantile, the distance (log2 scaled) of the TSSs to the nearest Ensembl gene TSS that has *protein_coding* biotype was calculated. Non-coding TSSs were divided according to the coding expression quantiles.

Relative contribution of pseudogenes to the assembly

All pseudogenes were retrieved from Ensembl mouse (v81); identified according the Ensembl pseudogene biotype definitions. Coding and non-coding transcript clusters with strand specific overlaps to unfiltered pseudogenes were determined using the GenomicRanges package in R. To remain conservative an overlap of 1+ nucleotides between assembly loci and pseudogenes was accepted.

lncRNA classification

lncRNAs were classified with respect to the protein coding features of ENSEMBL v81 and enhancer annotations. A lncRNA and a feature are considered to overlap where one or more exonic base is shared between the lncRNA and the feature. Promoters are defined as the region 1kb upstream of the annotated TSS. Enhancer data was retrieved from:
ftp://ftp.ebi.ac.uk/pub/databases/vertebrategenomics/FOG15/combinedPeakCalls/Mmus_H3K4me3.H3K27Ac_overlap_H3K27Aconly

For each of these categories, except for the intergenic and the enhancer overlapping lncRNAs, the orientation of the overlap is also shown with *sense* indicating an overlap in the same orientation and *antisense* indicating that the overlap is in the reversed orientation. If the lncRNA overlaps features on both strands they are labelled *Both*. Transcripts are labelled in a mutually exclusive fashion, following the ranking: TSS > TES > exon > intron > promoter > enhancer > intergenic.

Assembly comparisons

The assembly was compared to Ensembl (v81) annotations. Annotations mapping to chromosome patches were discarded. Initially transcriptome and annotations were converted to BED format using the UCSC tools *gtfToGenePred* and *genePredToBed*. Subsequently, the comparisons were performed with *BEDtools2 intersect* version 2-2.24.0. Overlaps are considered between features on the same strand. “1nt” reports the number of transcripts sharing at least a 1nt exonic overlap with Ensembl v81 features. The columns “10%” and “50%” indicate the minimum overlap (respectively 10% and 50%) required as a fraction of transcriptome assembly. For lncRNA cluster comparisons, Ensembl genes with the following biotypes are classed as long non coding genes: *3prime_overlapping_ncrna, ambiguous_orf, antisense, antisense_RNA, lincRNA, ncrna_host, non_coding, non_stop_decay, processed_transcript, retained_intron, sense_intronic, sense_overlapping*.

Repeat annotation

Unless otherwise stated, repeat annotation was derived using RepeatMasker (open-4.0.2) (`-engine ncbi -gff`) and NCBI/RMBLAST (v2.2.27+) and the RepeatMasker database of consensus elements (v20130422), run against individual chromosome sequences [28]. For ERV centric analyses redundancy is removed from the ERV annotation. The overlapping ERV with the lowest score is trimmed until no overlap remains. In a number of rare cases (196) where overlapping repeats have an equal score, both are retained. Where a repeat Bowtie index is required, repeat sequences are retrieved using *bedtools getfasta*. Species specific repeat annotation was also generated in this way for *mouse*, *rat* and *zebrafish*. Repeat elements, including ERV elements and LTRs, were classified according to their Repeatmasker defined consensus.

Promoter regions

Promoters were defined as the 1kb upstream of a TSS. Unless otherwise stated the relationship between ERV elements, promoters and TSSs was determined using the *GenomicRanges* R package.

ERV-subfamily classification based on enrichment in non-coding promoter regions

Unless stated, a non-redundant promoter set was used. To this end a random transcript was selected for each cluster, promoter regions were defined as above and all overlapping promoters were subsequently removed. ERVs selected for further analysis were associated with more than 50 promoter regions. Promoters where the maximum PhyloCSF score of the TSS derived transcripts was less than 50 were assigned as non-coding.

Analysis of ERV associated CAGE data

FANTOM5 CAGE data [29] was retrieved from the ENA [30] in FASTQ format (Samples: DRR009800, DRR009801, DRR009809, DRR009815, DRR009827, DRR009828, DRR009860, DRR009862, DRR009918, DRR009919, DRR009923, DRR009924, DRR009928, DRR009940, DRR009949, DRR009950, DRR009951, DRR009957, DRR009958, DRR009974, DRR009975, DRR009976, DRR009981, DRR009982, DRR009984, DRR010002, DRR010003 and DRR010005). *Tally*, a tool from the Kraken suite [7], was used to remove redundancy from the sequence files and read depth was recorded in the read header (Options: `-l 20 -tri 40 -format '>trn_%I_x%C%n%R%n'`). *Swan*, a second Kraken tool, was used to compare non-redundant fastq files to a database of rRNA sequences (Options: `-identity 90 -index 6 --grepv --grep -n-seeds 2 -w-seeds 8`). rRNA sequences were retrieved from RNACentral [31] (October 2015, Qurery: *Taxonomy 10090, rna_type "rRNA"*). Reads that do not map to rRNA sequences were aligned to a repeat masked genome generated in a chromosome-wise manner (RepeatMasker, NCBI/RMBLAST, Database: 20130422) with Bowtie allowing up to 3 mismatches (Options: `-k 1 -v 3 --time -f`). The number of genome mapping reads were retained for scaling purposes. Reads that did not match the genome were retained. These reads were mapped with Bowtie to an index of non-redundant ERV sequences (see above) allowing 2 mismatches (Options: `-v 2 --best --strata -a -S -f`). The reads were converted to BAM format and sorted with samtools. The number times each read mapped to the ERV-element file was counted and the depth of the read, recorded at the tally step, was divided between the number of alignments equally. An expression count in relation to each ERV-element was calculated from the split read depth of the associated alignments for both multi-mapping and uniquely mapping CAGE tags. Unless otherwise stated multimapping tag counts were used for repeat expression.

Expression heat maps were plotted using the R package *pheatmap*. Scaled repeat expression is scaled according to the number of CAGE tags mapping to the masked genome within each sample adjusted towards the sample with the maximum count.

Analysis of ERV-element CAGE tag orientation for the adult testes sample (DRR009975) was performed in R. ERV aligned BAM files were handled using the

ShortRead [32] and *Rsamtools* packages. ERV-elements not overlapping the promoter were discarded. For each ERV-element only the closest TSS pair was considered. Where an element overlaps multiple TSSs, the pair with the maximum overlap is retained. ERV mapped CAGE tag orientation was determined relative to the closest TSS. TSSs were assigned a coding potential based on the maximum phyloCSF score and minimum blast e-value of associated transcripts as described above. Within the heatmap the expression considers only uniquely mapped reads and is taken as the total $\log_2$(CAGE counts +1) sum averaged across the number of elements.

Genomic Alignment of reads for downstream analyses

All samples were aligned to the genome with TopHat2 [8]. The individual processed samples used for the transcriptome assembly were renamed and numbered and aligned against the mouse genome (mm10) as above. In the case of samples generated for subsequent evolutionary analysis mouse samples were aligned to mm10, rat samples were aligned to *Rnor_6.0*, zebrafish samples were aligned to *GRCz10*. genomes (Options: `--library-type fr-secondstrand --mate-inner-dist -50 -p 8`).

Assembly expression profiles using HTSeq

Aligned reads were sorted by name with samtools [33] (Options: *sort -n*) and converted to cluster-wise read counts using HTSeq [34] (Options: *--stranded=yes*). Mouse alignments were compared to the transcriptome assembly presented in this study. Rat and zebrafish reads were compared to their corresponding Ensembl annotation files. Corresponding annotation GTF files were retrieved from Ensembl (v81: Rattus_norvegicus.Rnor_6.0.81.gtf and Danio_rerio.GRCz10.81.gtf).

In order to generate the assembly expression heatmap, cluster-wise read counts were normalised using the *DESeq2* R package [35] and transformed using the variance stabilizing transformation. Clusters were classified as previously described and heatmaps were plotted using the *gplots* R package. For each cluster the median expression of each cell type was determined using all the samples presented in this study.

Determining the impact of promoter and TSS associated ERV on lncRNA gene expression

Transcripts were compared to a non-redundant set of ERV annotations (see above). TSSs were defined as the first nucleotide of each transcript while promoters corresponded to the 1000bp upstream. Where promoters extended beyond chromosome ends they were trimmed. Overlaps on either strand between ERVs, promoters and TSSs were determined with GenomicRanges in R. Genes were assigned to either TSS or Promoter overlaps sets if one or more one or more associated promoter

or TSS overlapped an ERV. Genes were preferentially assigned to the TSS set to remove redundancy. Gene expression was determined using HTSeq and converted to FPKMs (see below). Associated gene expression for each cell type was an average of the 2 appropriate samples.

ERV element expression gradients

Tophat2 aligned reads (see above) were filtered to remove multimappers using samtools [33] (Options: *-q50*) and to retain only the first segment in each read pair (`samtools view -f 64`, version 1.3). We then converted each of the filtered BAM files to two bigWig files containing reads mapping to the plus or minus strand respectively. This conversion was done in a three-steps process consisting of 1) BAM to BED conversion (bedtools bamToBed, v2.25.0); 2) BED to BedGraph conversion (bedItemOverlapCount); 3) BedGraph to bigWig (bedGraphToBigWig) [27]. We used computeMatrix (v2.2.3, *deepTools* suite [18]), to calculate the coverage on the positive and negative strands of repeat elements belonging to the selected sub-families previously described and within a 1Kb promoter region. We calculated the coverage on a region centred on the repeat (scaled to a uniform length of 5000bp) and encompassing 2000bp upstream and downstream. Repeats were split into those directly overlapping a TSS and repeats found elsewhere in the 1Kb promoter region. The resulting coverage matrices were imported into R for plotting. Briefly, each matrix was divided by a factor proportional to the total number of mapped reads in the corresponding BAM file and then log-transformed (after adding to each element the smallest non-zero value of its matrix). Subsequently, we used these matrices to produce coverage heatmaps and coverage profiles in the following way. To produce the heatmaps, we used ggplot2 (v2.1.0) to plot for each promoter (i.e. each row of the coverage matrix) the normalised, log-transformed coverage of each bin (i.e. each column of the coverage matrix). To produce the profile plots we calculated the mean coverage (as well as the standard error of the mean) of each bin across all repeat elements and flanking regions in the set and calculated a linear fit of the coverage as a function of the bin number within the repeat element itself.

Genome-wide non-coding promoter region enrichment and evolutionary conservation of repeat driven lncRNAs

In all cases, a redundant, complete repeat set was used for comparison. Repeats corresponding to the classes: *Simple repeat*, *Low complexity*, *snRNA*, *tRNA*, *rRNA*, *scRNA* and *srpRNA,* were removed prior to the analysis. Repeats on contigs not classified by Ensembl as a chromosome were removed. Repeats were organised according to 3 categories. In the first set repeats were grouped according to both their repeat sub-family and RepeatMasker family. In the second, repeats were divided according to their RepeatMasker family alone. Finally, repeats were divided based solely

on their class. In all cases within the groupings in each set, overlapping repeats were merged into a single interval to remove redundancy.

In the case of the mouse samples, analysis was performed relative to the assembly presented here. Genes and transcript clusters not found on Ensembl chromosomes were removed from the analysis. Gene clusters with ambiguous coding potential (eg. failed either blast or phyloCSF criteria) were discarded.

Expression profiles were generated using HTSeq as described above. Counts were converted to FPKMs considering the unique nucleotide coverage for each gene or cluster of transcripts and the total expression per sample. For each sample the average normalised expression between replicates was determined. For a gene to be considered 'expressed' in a cell type or tissue a minimal mean expression of 0.25 FPKM was set. Only genes with an expression greater than or equal to the cut-off were retained. For retained genes, the region 1kb promoter was obtained for each of their TSSs. Promoters overhanging chromosome ends were trimmed, strand information was removed and overlapping promoters were merged into a non-redundant set of promoter regions. These regions were assigned a biotype depending upon that of their parent gene clusters. Regions with both *coding* and *non-coding* (lncRNA) biotypes were labeled *ambiguous*. Coding, non-coding and ambiguous promoter regions were overlapped with the repeat sets and the overlaps with each repeat grouping were counted. Repeats sets with fewer than 50 promoter region overlaps were ignored. The ratio of *non-coding/coding* promoter regions was calculated for each repeat set together with the counts and ratio for genome wide promoter regions. For each set a one-tailed hypergeometic p-value was calculated, with the alternative hypothesis that promoter regions associated with repeat elements are more likely to be non-coding than expected.

In order to test the conservation of this repeat associated phenomenon in multiple species, the additional matched mouse, rat and zebrafish samples (described above) were used. Expression profiles were generated with HTSeq [34] as described above. For both rat and zebrafish samples Ensembl annotation was used in the place of the assembly. In these cases, annotated transcripts shorter than 200 nucleotides and with fewer than 2 exons were removed. Following Ensembl convensions: *3prime_overlapping_ncrna*, *ambiguous_orf*, *antisense*, *antisense_RNA*, *lincRNA*, *ncrna_host*, *non_coding*, *non_stop_decay*, *processed_transcript*, *retained_intron*, *sense_intronic*, *sense_overlapping;* gene biotypes were classed as *non-coding* whereas *protein_coding* were selected as *coding* genes. Genes from other categories were removed from further analysis.

The most enriched repeat families from the LTR, LINE and DNA classes were considered for coverage gradient analysis. In these cases, overlapping repeat elements in the same sets were merged and then overlapped with 1000bp regions upstream of a non-redundant set of genome-wide TSSs. Promoters containing a repeat element were paired with the coordinates of the associated repeat.

Genomewide enrichment of ERVs in promoters

For rat and zebrafish, Ensembl annotations (v81) were used, filtered to remove short and non-spliced transcripts and separated according to coding potential as above. Manipulation of genomic regions was performed using the GenomicRanges package in R. Only annotation on Ensembl specified *chomosomes* are considered. Effective genome size was determined by counting the total number of A, T, G and C nucleotides on these chromosomes (effectively removing N's). Cell type FPKM expression was determined using HTSeq counts as described above and averaged across sample duplicates. Redundant repeat datasets were used for the determination of enrichments. Repeats with the following classes were removed: *Simple_repeat, Low_complexity, snRNA, tRNA, rRNA, scRNA* and *srpRNA*. Repeats were all considered to be strand non-specific. When considering all repeat classes or repeat families, the number of repeats in each repeat subset (eg. a class) were counted and subsets were discarded where the number of repeats in the set fell below the median across all sets. Within each set, redundancy was removed as overlapping repeats were collapsed to combined regions. Finally for all of the collapsed repeat regions only the central nucleotide was taken as representative. In each sample type, genes or assembly transcript clusters were filtered according to a series of minimum expression thresholds and all transcripts from clusters below each threshold were discarded. For the remaining transcripts 2 sets of 5' regions were defined; promoter regions of 1kb upstream of each TSS and TSS regions of 400nt with the TSS at the centre. Regions were trimmed if they extended beyond the chromosome boundaries. Each set of 5' regions was processed sequentially. Regions corresponding to non-coding genes were retained, strand information was removed and overlapping regions were merged into combined genomic spans. Subsequently the number of bases represented by the complete set of 5' regions was calculated. For each set of repeats to be tested, the number of instances where a central repeat nucleotide falls within one of the 5' regions was counted. Given an alternative hypothesis that repeat elements are enriched in the 5' regions of non-coding transcripts, a binomial test was performed to determine if a significant enrichment could be detected. The genome size and bases represented by the 5' regions were used to estimate the probability of a repeat falling within a promoter region, and the genome-wide number of repeat regions and the number of repeats in the promoter regions represented the number of trials and successes respectively. P-values for enrichment were adjusted according to the number of repeat sets tested according to the Hochberg (1988) method.

Sequence based relationship between ERV-elements

To illustrate the landscape of sequence similarities for the ERV-element sequences, we took all 581 sequences of type 'LTR' from the RepeatMasker library (v20130422) and blasted them against each other with default parameters. A normalised similarity score between each pair of sequences was defined as the blast bit score, divided by the larger of the two self-bit-scores. The resulting network structure was clustered using MCL [36] with default parameters. The list of 17 sequences of interest was augmented by adding the additional elements from the MCL clusters in which they were found, leading to a total set of 54 sequences. Clustering within the heatmap was performed using the standard R clustering hclust with complete linkage clustering. For the hclust clustering the distance measure used was the normalised score described above subtracted from one.

Longest open reading frame analysis

The longest, complete ORF is selected for each transcript in any frame. For cluster based analysis, where a transcript possesses multiple ORFs of the same length a random ORF was selected. Subsequently, if multiple transcripts from the same cluster possess a 'longest ORF' of equal length, again a random selection is made to choose a representative. PhyloP scores, corresponding to mm10, from the 60 vertebrate alignment were retrieved from UCSC. For each transcript the proportion of significant positive (conserved) and negative (rapidly evolving) PhyloP scores for the each of the representative ORFs and respective UTRs was calculated (cut off = 1.301). Where PhyloP scores from within a transcript were missing this calculation was skipped.

For ORF and UTR phyloP conservation analysis, clusters with either a longest ORF or 3'UTR length less than 50 bases were removed in addition to ORFs and 3'UTRs associated with missing phyloP scores. Density contours were calculated with the kde2d function in the *MASS* R package. Significance was calculated by one-sided sign test following the alternative hypothesis that ORFs have a greater proportion of rapidly evolving bases than the concurrent 3'UTR. Where overlaps are removed, all overlapping clusters are disregarded for the sign test calculation. "Poorly conserved" clusters were selected by considering only cluster ORFs where the proportion of ORF bases with a significant, positive PhyloP score + the proportion of the paired 3'UTR bases with a significant, positive PhyloP score < 0.4.

Gene Ontology analysis

The transcripts selected to represent the longest ORF for each coding cluster were overlapped with the transcripts of "protein_coding" Ensembl genes. Transcripts were paired with any overlapping Ensembl genes and those transcripts associated with

multiple genes were discarded. Transcripts with shared Ensembl annotation were also discarded. Transcripts without complete phyloP information for both the ORF and 3'UTR were also discarded along with transcripts for which both the ORF and 3'UTR are not equal to or more than 50nt in length. The Ensembl set representing the remaining transcripts was designated as the background. Non-conserved transcripts were selected as above. Rapidly evolving and slowly evolving, non-conserved ORFs were demarked according to whether the proportion of bases in their ORF with a significant, negative phyloP score minus the respective proportion of the corresponding UTR is more than or less than 0. GO enrichment analysis was performed using the GOrilla online web service [37], using the two list mode in *Mus musculus*. The GOrilla database was updated on 6th August 2016.

Mouse ENCODE tissue-specific ERV analysis

Paired-end strand-specific RNASeq from 13 ENCODE primary mouse tissues was obtained from www.encodeproject.org (biological replicates). (Accessions: ENCSR288TLO,ENCSR713OCQ,ENCSR554PHF,ENCSR164BAZ,ENCSR394YLM,ENCSR216KLZ,ENCSR870AQU,ENCSR516UNF,ENCSR248XKS,ENCSR518GDK,ENCSR170SVO,ENCSR966JPL,ENCSR266ESZ). These files were mapped against the Mouse genome (GRCm38) using hisat2 with the "—fr" flag to specify strand-specific paired-end reads. Hisat2 output was converted to bam format using samtools and htseq-count was used (with option: --stranded=yes) to generate count tables according to version 87 of the Ensembl GTF annotation file for mouse (Mus_musculus.GRCm38.87.gtf). These counts were loaded into R/BioConductor and normalised using DESeq2. A tissue-specificity score τ was calculated for each transcript according to the following equation: $\tau = \frac{\sum_{i=1}^{N}(1-x_i)}{N-1}$. Where N is the number of tissues and x_i is the expression profile component normalised by the value of the maximum component [38]. Transcripts whose specificity score exceeded a threshold ($\tau >= 0.75$) were deemed tissue-specific and flagged according to the tissue of their highest number of normalised counts. The TSS site and upstream 1000bp were computed from the GTF annotation (above) for each tissue-specific transcript. Each tissue-specific transcript was also deemed as either protein coding or a lncRNA according to Ensembl biotype flags. When multiple transcripts occupied the same TSS site or promoter region only the first was selected to avoid redundancy. These TSS regions were then compared to a BED file containing ERV locations in the mouse genome to determine which directly overlap an annotated ERV. When multiple ERVs were deemed to overlap, the one with the highest score was taken. Where necessary expression levels were converted to FPKMs (see Appendix figures).

Mass spectroscopy

Non-coding cluster candidates were selected for targeted mass spectroscopy based on a series of criteria:

1. For each non-coding cluster a transcript associated with the longest ORF was selected in each case as described above.
2. The TSS of the transcript must overlap an ERV-element.
3. The associated cluster must be most highly expressed in either spermatocytes or round spermatids as determined by RSEM FPKM values.
4. Selected ORFs must represent clusters expressed within the top 50% of all clusters, ordered by their maximum expression in either spermatocytes or round spermatids.
5. ORFs must not represent clusters that have multiple ORFs of equal length to the chosen ORF, but which code for different peptides.
6. ORFs representing clusters with a $-\log_{10}$(BLASTx E-value) >0 (see above) were removed.
7. For each ORF a p-value was generated to express the likelihood that the longest ORF has been identified by chance within its representative transcript. Transcripts were shuffled 10000 times using ushuffle (Options: *-k 2*). In each case the longest ORF was identified. An extreme value distribution was fitted to model the ORF length and a p-value ascribed to the length of "true" ORF using the *evd* and *extRemes* R packages. Candidate ORFs were ordered by P-value.

The ORFS selected were used to create a database for LC MS/MS search. Germ cells were stained and purified as described, sorted spermatocytes and round spermatids were spun down at 3000 for 10 minutes at 4°C, the pellet was washed twice with cold PBS and proteins were extracted on ice with extraction buffer (Tris pH 8.0 50 mM, NaCl 150 mM. MgCl2 5 mM, DTT 1mM, SDS 2%) added with proteinase inhibitors. Extracts were passed several times though an insulin syringe and solution was finally cleared with 20 minutes of centrifugation at max speed at 4°C [39]. Peptides (200ng) were injected on EasySpray 50cm column (Thermo) connected to Orbitrap Fusion Lumos (Thermo) for data-dependent experiment. Raw files were searched using Mascot (Matrix Biosciences) with the following settings: MS1 tolerance: 5ppm, MS2: 0.1Da, max missed cleavages: 2. Samples were re-analysed with the mass spectrometer operated in targeted mode, when only ERV-derived peptides selected from previous experiment were measured. Targeted data extraction (MS1 filtering and PRM) was performed using Skyline 3.5 [40] with tolerances of dotp>0.75, idotp>0.8.

REFERENCES

1. Dolznig H, Boulme F, Stangl K, Deiner EM, Mikulits W, Beug H, Mullner EW (2001) Establishment of normal, terminally differentiating mouse erythroid progenitors: molecular characterization by cDNA arrays. *FASEB J* **15**: 1442-4

2. von Lindern M, Deiner EM, Dolznig H, Parren-Van Amelsvoort M, Hayman MJ, Mullner EW, Beug H (2001) Leukemic transformation of normal murine erythroid progenitors: v- and c-ErbB act through signaling pathways activated by the EpoR and c-Kit in stress erythropoiesis. *Oncogene* **20**: 3651-64

3. Kanatsu-Shinohara M, Ogonuki N, Inoue K, Miki H, Ogura A, Toyokuni S, Shinohara T (2003) Long-term proliferation in culture and germline transmission of mouse male germline stem cells. *Biol Reprod* **69**: 612-6

4. Barchi M, Geremia R, Magliozzi R, Bianchi E (2009) Isolation and analyses of enriched populations of male mouse germ cells by sedimentation velocity: the centrifugal elutriation. *Methods Mol Biol* **558**: 299-321

5. Bastos H, Lassalle B, Chicheportiche A, Riou L, Testart J, Allemand I, Fouchet P (2005) Flow cytometric characterization of viable meiotic and postmeiotic cells by Hoechst 33342 in mouse spermatogenesis. *Cytometry A* **65**: 40-9

6. Kimmel CB, Ballard WW, Kimmel SR, Ullmann B, Schilling TF (1995) Stages of embryonic development of the zebrafish. *Dev Dyn* **203**: 253-310

7. Davis MP, van Dongen S, Abreu-Goodger C, Bartonicek N, Enright AJ (2013) Kraken: a set of tools for quality control and analysis of high-throughput sequence data. *Methods* **63**: 41-9

8. Trapnell C, Roberts A, Goff L, Pertea G, Kim D, Kelley DR, Pimentel H, Salzberg SL, Rinn JL, Pachter L (2012) Differential gene and transcript expression analysis of RNA-seq experiments with TopHat and Cufflinks. *Nat Protoc* **7**: 562-78

9. Haas BJ, Papanicolaou A, Yassour M, Grabherr M, Blood PD, Bowden J, Couger MB, Eccles D, Li B, Lieber M*, et al.* (2013) De novo transcript sequence reconstruction from RNA-seq using the Trinity platform for reference generation and analysis. *Nat Protoc* **8**: 1494-512

10. Derti A, Garrett-Engele P, Macisaac KD, Stevens RC, Sriram S, Chen R, Rohl CA, Johnson JM, Babak T (2012) A quantitative atlas of polyadenylation in five mammals. *Genome Res* **22**: 1173-83

11. Altschul SF, Gish W, Miller W, Myers EW, Lipman DJ (1990) Basic local alignment search tool. *J Mol Biol* **215**: 403-10

12. Lin MF, Jungreis I, Kellis M (2011) PhyloCSF: a comparative genomics method to distinguish protein coding and non-coding regions. *Bioinformatics* **27**: i275-82

13. Speir ML, Zweig AS, Rosenbloom KR, Raney BJ, Paten B, Nejad P, Lee BT, Learned K, Karolchik D, Hinrichs AS*, et al.* (2016) The UCSC Genome Browser database: 2016 update. *Nucleic Acids Res* **44**: D717-25

14. Finn RD, Coggill P, Eberhardt RY, Eddy SR, Mistry J, Mitchell AL, Potter SC, Punta M, Qureshi M, Sangrador-Vegas A*, et al.* (2016) The Pfam protein families database: towards a more sustainable future. *Nucleic Acids Res* **44**: D279-85

15. Wang L, Park HJ, Dasari S, Wang S, Kocher JP, Li W (2013) CPAT: Coding-Potential Assessment Tool using an alignment-free logistic regression model. *Nucleic Acids Res* **41**: e74

16. Li B, Dewey CN (2011) RSEM: accurate transcript quantification from RNA-Seq data with or without a reference genome. *BMC Bioinformatics* **12**: 323

17. Pollard KS, Hubisz MJ, Rosenbloom KR, Siepel A (2010) Detection of nonneutral substitution rates on mammalian phylogenies. *Genome Res* **20**: 110-21

18. Ramirez F, Dundar F, Diehl S, Gruning BA, Manke T (2014) *deepTools*: a flexible platform for exploring deep-sequencing data. *Nucleic Acids Res* **42**: W187-91

19. Derrien T, Johnson R, Bussotti G, Tanzer A, Djebali S, Tilgner H, Guernec G, Martin D, Merkel A, Knowles DG*, et al.* (2012) The GENCODE v7 catalog of human long noncoding RNAs: analysis of their gene structure, evolution, and expression. *Genome Res* **22**: 1775-89

20. Esteve-Codina A, Kofler R, Palmieri N, Bussotti G, Notredame C, Perez-Enciso M (2011) Exploring the gonad transcriptome of two extreme male pigs with RNA-seq. *BMC Genomics* **12**: 552

21. Gish W (1996) In

22. Slater GS, Birney E (2005) Automated generation of heuristics for biological sequence comparison. *BMC Bioinformatics* **6**: 31

23. Taly JF, Magis C, Bussotti G, Chang JM, Di Tommaso P, Erb I, Espinosa-Carrasco J, Kemena C, Notredame C (2011) Using the T-Coffee package to build multiple sequence alignments of protein, RNA, DNA sequences and 3D structures. *Nat Protoc* **6**: 1669-82

24. Smit AFAH, R.; Green, P. (2015) RepeatMasker Open-4.0.2013-2015 In

25. Mouse Encode Consortium, Stamatoyannopoulos JA, Snyder M, Hardison R, Ren B, Gingeras T, Gilbert DM, Groudine M, Bender M, Kaul R*, et al.* (2012) An encyclopedia of mouse DNA elements (Mouse ENCODE). *Genome Biol* **13**: 418

26. Langmead B, Salzberg SL (2012) Fast gapped-read alignment with Bowtie 2. *Nat Methods* **9**: 357-9

27. Quinlan AR, Hall IM (2010) BEDTools: a flexible suite of utilities for comparing genomic features. *Bioinformatics* **26**: 841-2

28. Bao W, Kojima KK, Kohany O (2015) Repbase Update, a database of repetitive elements in eukaryotic genomes. *Mob DNA* **6**: 11

29. Abugessaisa I, Shimoji H, Sahin S, Kondo A, Harshbarger J, Lizio M, Hayashizaki Y, Carninci P, consortium F, Forrest A*, et al.* (2016) FANTOM5 transcriptome catalog of cellular states based on Semantic MediaWiki. *Database (Oxford)* **2016**

30. Leinonen R, Akhtar R, Birney E, Bower L, Cerdeno-Tarraga A, Cheng Y, Cleland I, Faruque N, Goodgame N, Gibson R*, et al.* (2011) The European Nucleotide Archive. *Nucleic Acids Res* **39**: D28-31

31. Bateman A, Agrawal S, Birney E, Bruford EA, Bujnicki JM, Cochrane G, Cole JR, Dinger ME, Enright AJ, Gardner PP*, et al.* (2011) RNAcentral: A vision for an international database of RNA sequences. *RNA* **17**: 1941-6

32. Morgan M, Anders S, Lawrence M, Aboyoun P, Pages H, Gentleman R (2009) ShortRead: a bioconductor package for input, quality assessment and exploration of high-throughput sequence data. *Bioinformatics* **25**: 2607-8

33. Li H, Handsaker B, Wysoker A, Fennell T, Ruan J, Homer N, Marth G, Abecasis G, Durbin R, Genome Project Data Processing S (2009) The Sequence Alignment/Map format and SAMtools. *Bioinformatics* **25**: 2078-9

34. Anders S, Pyl PT, Huber W (2015) HTSeq--a Python framework to work with high-throughput sequencing data. *Bioinformatics* **31**: 166-9

35. Love MI, Huber W, Anders S (2014) Moderated estimation of fold change and dispersion for RNA-seq data with DESeq2. *Genome Biol* **15**: 550

36. van Dongen S (2008) Graph clustering via a discrete uncoupling process. *Siam Journal on Matrix Analysis and Applications* **30**: 121-141

37. Eden E, Navon R, Steinfeld I, Lipson D, Yakhini Z (2009) GOrilla: a tool for discovery and visualization of enriched GO terms in ranked gene lists. *BMC Bioinformatics* **10**: 48

38. Yanai I, Benjamin H, Shmoish M, Chalifa-Caspi V, Shklar M, Ophir R, Bar-Even A, Horn-Saban S, Safran M, Domany E*, et al.* (2005) Genome-wide midrange transcription profiles reveal expression level relationships in human tissue specification. *Bioinformatics* **21**: 650-9

39. Wisniewski JR, Zougman A, Nagaraj N, Mann M (2009) Universal sample preparation method for proteome analysis. *Nat Methods* **6**: 359-62

40. MacLean B, Tomazela DM, Shulman N, Chambers M, Finney GL, Frewen B, Kern R, Tabb DL, Liebler DC, MacCoss MJ (2010) Skyline: an open source document editor for creating and analyzing targeted proteomics experiments. *Bioinformatics* **26**: 966-8
